# Toxic Metals in a Green Transition: Global Health Risks, Sources, and Policy Responses—Insights from the Munich Toxic Metals Symposium 2025

**DOI:** 10.5334/aogh.5214

**Published:** 2026-04-07

**Authors:** Stephan Bose-O’Reilly, Stefan Rakete, Philip J. Landrigan, Johanna Elbel, Monica Nordberg, Gunnar Nordberg, Karin Broberg, Dewi Yunia Fitriani, Jenna Forsyth, Joanna Gaitens, Jinky Leilanie Lu, Dennis Nowak, Ernesto Sanchez-Triana, Sophie Turner, John Yabe, Melissa McDiarmid, Florencia Harari

**Affiliations:** 1 Institute and Clinic for Occupational, Social, and Environmental Medicine, University Hospital, LMU Munich, Ziemssenstr. 5, D-80336 Munich, Germany; 2 Global Observatory on Planetary Health, Boston College, Chestnut Hill, MA, USA; 3 Center Scientifique de Monaco, Monaco; 4 School of Public Affairs, Sciences Po University, 1 place Saint-Thomas d’Aquin, 75007, Paris, France; 5 Karolinska Institutet, Stockholm, Sweden; 6 Umea University, Umea, Sweden; 7 Lund University, Lund, Sweden; 8 Occupational and Environmental Health Research Centre, Indonesian Medical and Education Research Institute (IMERI), Faculty of Medicine, Universitas Indonesia, Central Jakarta, Indonesia; 9 Stanford University, Stanford, CA, USA; 10 University of Maryland School of Medicine, Baltimore, USA; 11 National Institutes of Health, University of the Philippines, Manila, Philippines; 12 World Bank, Washington DC, USA; 13 Leigh Day, London, UK; 14 University of Namibia, Windhoek, Namibia; 15 Occupational and Environmental Medicine, School of Public Health and Community Medicine, University of Gothenburg and Sahlgrenska University Hospital, Sweden

**Keywords:** green transition, critical raw materials, lithium, cobalt, nickel, lead, mercury, uranium, health, policy interventions, occupational & environmental safety

## Abstract

*Background:* The global energy transition toward climate neutrality is driving rapid growth in the demand for critical minerals such as lithium, cobalt, and nickel. While indispensable for decarbonization, their extraction, processing, and recycling expose workers, communities, and ecosystems to toxic metals, including lead, mercury, arsenic, cadmium, and manganese, raising significant public health concerns.

*Objectives:* This article synthesizes evidence presented at the Toxic Metals Symposium 2025 in Munich to assess health impacts, exposure pathways, and policy challenges related to toxic metals in the green energy transition.

*Methods:* The analysis integrates findings from multidisciplinary studies presented at the symposium, including environmental monitoring, biomonitoring, occupational health research, and policy assessments across multiple geographic contexts.

*Findings:* Evidence from mining regions, informal recycling hubs, and urban areas demonstrates widespread and persistent exposure to toxic metals from both legacy and ongoing sources. Lead and mercury are linked to impaired cognitive development in children, cardiovascular disease, kidney failure, hypertension, and anemia. Arsenic and cadmium exposures are associated with increased cancer risk and renal dysfunction. Occupational studies report cobalt and nickel exposures exceeding safety thresholds, even in modern battery recycling facilities. Emerging research highlights cumulative, low-dose, and transgenerational effects, particularly among vulnerable populations. Although advances in monitoring technologies and community biomonitoring improve detection, weak regulatory enforcement and the prevalence of informal sectors continue to limit risk reduction.

*Conclusions:* Managing toxic metal risks is essential for a just and sustainable green transition. Health safeguards must be embedded in climate and industrial policies from the outset, supported by enforced corporate due diligence and long-term remediation financing. Protecting human health must be a co-equal priority to carbon reduction to prevent reproducing historical environmental injustices.

## Background

### Green transition

The imperative to limit global warming to well below 2°C has accelerated a worldwide shift toward clean energy technologies and sustainable economic systems. Renewable energy generation, electrification of transport, and large‑scale energy storage are central pillars of this transition, driving an unprecedented demand for critical minerals and metals such as lithium, cobalt, nickel, and various rare earth elements. Projections by the International Energy Agency estimate that the demand for key minerals may increase fourfold by 2040 to meet the targets of the Paris Agreement, which was adopted in 2015.^1^ This surge poses unique challenges for mineral‑rich but economically vulnerable countries, where extraction and processing often occur under weak regulatory frameworks.

### Toxic minerals and metals (old and new)

While the green transition aims to mitigate the catastrophic impacts of climate change, it paradoxically risks aggravating environmental and public health threats through increased toxic metal emissions and exposure. Extraction, refining, manufacturing, recycling, and improper disposal of these minerals can release the “classic” toxic metals such as lead (Pb), mercury (Hg), arsenic (As), cadmium (Cd), and chromium (Cr) into air, soil, and water systems. Nickel (Ni) and cobalt (Co) are further potentially harmful metals. These metals are non‑biodegradable and persist in ecosystems. They also bioaccumulate in food chains. There is well‑documented research demonstrating the cardiovascular, neurological, carcinogenic, reproductive, and multi‑organ effects that they exert in humans.

### Toxic metals symposium

A Toxic Metals Symposium was held from July 4 to July 5, 2025, at the Ludwig Maximilian University (LMU), Munich, Germany. It was organized by the Institute and Clinic for Occupational, Social and Environmental Medicine at the University Hospital, LMU, Munich, and the Collegium Ramazzini. There were 31 oral presentations and 13 poster presentations. Therein total were over 150 participants, including researchers, policy‑makers, public health practitioners, industry representatives, and community stakeholders, from at least 50 countries. Speakers at the *Toxic Metals Symposium 2025* repeatedly stressed this double burden: achieving net‑zero carbon emissions must not come at the cost of heightened toxic metal exposure for workers and communities. In her keynote address, Karin Broberg (Lund University) argued that without integrated health safeguards, the pursuit of low‑carbon technologies risks repeating the legacy of environmental injustices associated with past mining booms [1]. Philip Landrigan (Boston College) and Ernesto Sanchez‑Triana (World Bank) reinforced this by highlighting lessons from the decades‑long global struggle to phase out leaded gasoline, a success story that required political will, international cooperation, and strong public health advocacy (see supplement AB Landrigan). Fortunately, the replacement of lead compounds with manganese compounds as fuel additives has been halted thanks to early warnings about neurotoxic effects in the Brescia Declaration [2] that may develop in a similar way for manganese as experienced with lead. All dispersive uses of toxic metals that contaminate our environment should be discouraged [2].

### Sources of toxic metals

Legacy pollution from historical mining, smelting, and industrial activities continues to endanger millions of people worldwide. Despite bans on leaded petrol and restrictions on mercury use under the Minamata Convention on Mercury, children and adults in many regions still live with dangerously elevated body burdens of the metals. The resurgence of mineral extraction to fuel green technologies risks adding new layers of exposure, especially in low‑ and middle‑income countries (LMICs) where regulatory oversight and remediation budgets are limited. Florencia Harari (University of Gothenburg) presented emerging data on cobalt and nickel exposure among battery recycling plant workers in the Nordic countries (see supplement AB Harari). Raw materials used to produce such batteries are mainly mined in the southern hemisphere under poor working conditions and with dramatic negative effects on the surrounding communities. This echoes concerns from Africa’s copper and cobalt belts shared by Paul Musa Obadia (University of Lubumbashi) (see supplement AB Musa Obadia).

Artisanal and small‑scale gold mining (ASGM), the largest source of global mercury emissions, exemplifies this tension. Driven by poverty, unemployment, and a significant increase in the gold price, millions depend on ASGM for livelihoods despite severe health risks [3]. Stephan Bose‑O’Reilly (LMU Munich) summarized multi‑country studies demonstrating that children living near ASGM sites show impaired neurodevelopment and high mercury levels that exceed World Health Organization (WHO) safety thresholds (see supplement AB Bose‑O’Reilly).

Further critical raw materials and rare‑earth elements are needed in the transition toward a circular economy free from fossil fuels. To decrease the need for mining, higher recycling of minerals is needed, part of which is done from e‑waste. Karin Broberg (Lund University) presented results from a Swedish study on recyclers showing higher blood and/or post‑shift urine concentrations of metals, including indium, compared to controls [4].

Adding complexity, the transition to a circular economy has boosted informal e‑waste recycling in LMICs [5]. Sites like the Agbogbloshie dumpsite in Ghana are notorious for uncontrolled burning and acid leaching, releasing lead, cadmium, and arsenic. Andrea Kaifie‑Pechmann (FAU Erlangen) and Johanna Elbel (Sciences Po Paris) documented the profound community exposure and social inequities intertwined with this sector (see supplement AB Elbel and AB Kaifie‑Pechmann). Even in high‑income settings, residual contamination persists: Lea John (LMU Munich) presented evidence of elevated blood lead among children in Goslar, Germany, a reminder that historical pollution can continue to pose health threats for generations (see supplement AB John).

### Policies

The Declaration of Brescia, adopted at a 2006 international workshop organized by the International Commission on Occupational Health at the University of Brescia, addressed the neurotoxicity of lead, mercury, and manganese [2]. It highlighted that serious subclinical and developmental brain damage occurs at exposure levels far below earlier assumptions, while preventive action has historically been delayed. The Declaration called for precautionary responses to early warnings, elimination of remaining lead uses, stricter exposure standards, reduced mercury emissions and dietary risks, and avoidance of manganese in gasoline. It emphasized that prevention costs are far lower than the lifelong social and economic burdens of neurotoxicity [2]. Building on this 2006 Declaration, as well as a previous Collegium Ramazzini statement calling for reduced disease and death from artisanal and small‑scale mining (ASM) [3].

The *Toxic Metals Symposium 2025* participants charted a path toward minimizing toxic metal hazards in the era of sustainable development.

The conclusion of the *Toxic Metals Symposium 2025* that protecting health must be a co‑equal priority alongside carbon reduction in the energy transition, particularly for communities bearing disproportionate environmental burdens, aligns with a recent ruling by the International Court of Justice. In 2025, the Court ruled that all UN Member States are bound by a collective responsibility to collaborate to ensure the preservation of the Earth’s environment.^2^ This ruling elevates the importance of health protection, environmental protection, and social justice in the Green Transition.

Addressing toxic metal contamination in the Green Transition is also integral to achieving multiple sustainable development goals (SDGs), including good health and well‑being (SDG 3), clean water and sanitation (SDG 6), decent work and economic growth (SDG 8), sustainable cities and communities (SDG 11), and responsible consumption and production (SDG 12). It also resonates with the principle of environmental justice, ensuring that the transition to sustainability does not exacerbate social inequalities but instead fosters health equity and ecological resilience worldwide.

### Synthesis

The shift in demand is altering global supply chains, expanding extraction into new regions, and increasing reliance on hazardous practices and informal workers. Toxic metal emissions are rising, particularly from ASM, including cobalt and gold extraction, as well as from the recycling of used lead‑acid batteries (ULABs) in LMICs. These activities, often conducted with limited oversight, pose significant health risks to workers and nearby communities. Importantly, such risks are present in all countries, even high‑income countries (HICs) [6]. However, LMICs, where informal recycling continues to cause widespread health and environmental harm, face the highest risks. While the hazards of cadmium, manganese, and mercury are well established, their burden of disease has not yet been comprehensively quantified. Even less is known about the health and environmental impacts of newer materials such as cobalt and lithium.

## Objective

This article examines how the global energy transition is reshaping demand for minerals, both conventional and emerging, focusing on critical raw materials required for renewable energy technologies.

Synthesizing the symposium’s key scientific insights and consensus recommendations, this article first reviews the contemporary and legacy sources of toxic metals and key exposure pathways (section “Objective”), summarizes major health effects (section “Health Hazards of Toxic Metals”), and highlights recent advances in exposure monitoring and risk assessment (section “Advances in Monitoring and Risk Assessment, Gaps”). Section “Policy and Governance Challenges” discusses policy and governance challenges, and section “Roadmap for a Safe and Equitable Green Transition” proposes a stepwise roadmap to safeguard communities while sustaining the mineral supplies essential for a decarbonized future. The final sections reflect on these findings and outline urgent actions for research, regulation, and community engagement.

### Sources of exposure to toxic metals

New industries driving the global energy transition have intensified demand for critical raw materials, disrupted supply chains, and increased exposure to toxic metals. At the same time legacy pollution from mining and informal industrial practices continues to pose pervasive risks to communities and workers.

Speakers at the *Toxic Metals Symposium 2025* illustrated how a combination of new extraction activities, legacy pollution, and tainted consumer products contribute to toxic metal exposure.

### Industrial mining and processing of critical minerals

To satisfy the demand for low‑carbon technologies, there has been a considerable increase in the extraction and processing of cobalt, nickel, lithium, copper, manganese, and rare‑earth elements. Mining activities are concentrated in South America, Southeast Asia, and Central Africa, regions where occupational and environmental safety measures are often inadequate [7, 8].

Around one‑third of global rare‑earth reserves are located in China, which produces 95% of the global supply. Recently, Europe’s largest reserves of rare‑earth elements were found in the north of Sweden.^3^ Rare‑earth elements are essential for electric vehicles, wind turbines, and electronics, playing a critical role in the green transition.

In the Congolese Copperbelt, artisanal and industrial activities are found to be intertwined. Paul Musa Obadia’s (University of Lubumbashi) research indicated that industrial copper smelter workers exhibited elevated levels of cobalt and lead in their blood, as well as increased urinary concentrations of germanium when compared to control groups. At the same time, artisanal miners’ reproductive health was affected [9].

A hierarchical structure is in place within the workforce, with large corporations prioritizing the protection of their own employees over others. In the context of subcontracting, workers are exposed to a heightened risk of hazardous conditions. Brandon Phathisani Sibanda (Enviro recsus) presented an assessment of the environmental impacts of mineral mining in Zimbabwe, focusing on policy gaps, investor practices, and community‑level activities. He highlighted that insufficient environmental legislation, limited investor accountability, and small‑scale mining contribute to environmental degradation and toxic metal exposure, while weak enforcement, particularly in projects run by foreign operators, reduces the country’s ability to mitigate harm (see supplement AB Sibanda).

Uranium mining takes place in countries such as Canada, Australia, Kazakhstan, and the United States, where large deposits of uranium ore are found. The process releases radioactive emissions, including radon gas and uranium dust, as well as toxic metals and other contaminants into air and water. These emissions can contribute to long‑term environmental pollution and pose health risks to nearby populations [10]. The excess of lung cancer observed in uranium miners has been attributed primarily to radon exposure reflecting occupational exposure during mining [11]. Uranium miners have been shown to experience dose‑related increases in lung cancer incidence and mortality that are magnified by cigarette smoking [12]. The possibility exists that uranium mining may increase in coming years as an unintended consequence of the Green Transition as the nuclear industry and its political supporters advance the false claim that nuclear energy represents a “clean, reliable, safe, and affordable” alternative to fossil fuels [13].

At the rear end of the supply chain, Florencia Harari (University of Gothenburg) presented pilot data on metal exposure through air, dermal contact, and biomonitoring at a new large‑scale battery recycling facility for lithium‑ion batteries (LiB) for electric vehicles in the Nordic countries (see supplement AB Harari). The airborne concentrations of nickel and cobalt, which are essential components of these batteries, were elevated and several fold higher than the occupational exposure limits for all workers in the separation and sorting areas. Direct reading particle instruments revealed elevated peak dust exposures during specific working tasks. In addition, nickel and cobalt were detected on the hands of all the examined workers.

In the coming decades, the demand for lithium, a mineral essential for modern batteries, is projected to rise dramatically, reaching an increase of up to 40 times its current levels. A substantial proportion of global lithium reserves, approximately 53%, are found in brines, defined as hypersaline aquifers where the accumulation of solutes, including lithium, has been driven by a prolonged process of evaporation that has outpaced precipitation over long periods of time. The majority of deposits in the “Lithium Triangle” region of South America are found in closed, or endorheic, basins. The dearth of contemporary precipitation‑based freshwater inflows, which are imperative to maintaining adequate water supplies for indigenous populations, rare plants and animals, and mining operations, constitutes a significant problem in the Lithium Triangle [14].

### Artisanal and small‑scale mining

Artisanal and small‑scale mining (ASM) is one of the main sources of toxic metal emissions, particularly mercury and lead. Laura Emilce Flores Rodríguez (Hospital de Clínicas) reported health impacts among 99 artisanal gold miners in Paso Yobai, Paraguay, where 61.4% handled mercury directly. Neurotoxic symptoms were prevalent, with 42.4% reporting memory disorders and 39.8% showing mild cognitive impairment on Mini Mental testing. Urinary mercury levels were significantly associated with direct mercury handling (*p* = 0.006) (see supplement AB Flores)). Stephan Bose‑O’Reilly (LMU Munich) showed that ASGM is the main global source of mercury pollution, contributing over 38% of global emissions [15]. Across sub‑Saharan Africa (SSA), including in Ghana, Tanzania, Kenya, Senegal, and Zimbabwe, it is the major source of regional mercury emissions (see supplement AB Bose‑O’Reilly). A study conducted in Zimbabwe revealed that artisanal and small‑scale gold miners in the mining towns of Kadoma and Shurugwi engaged in the practice of open amalgam burning and cyanide leaching from mercury‑containing tailings. This practice exposed the miners to mercury, cyanide, chemical dust, and gases [16]. Uncontrolled amalgamation and burning practices in SSA, South‑East Asia, and Latin America have resulted in miners and children having hair mercury levels of 10–15 mg/kg, which is far above the 1 mg/kg reference threshold [17].

In the Philippines, Jinky Leilanie Lu (University of the Philippines) showed that 8% of ASGM miners had blood mercury levels above 15 µg/L, which require intervention according to the Human‑Biomonitoring categories of the German Environmental Protection Agency. These results demonstrate that the risks of ASM are widespread and not limited to isolated communities (see supplement AB Lu JL). The repercussions of ASM are not confined to terrestrial environments; they also manifest in marine ecosystems. Two case studies presented by Omar Keshk (GEOMAR Helmholtz Centre for Ocean Research), one from a site near the Magdalena River mouth in Colombia and the other from Papua New Guinea, indicated that both artisanal and industrial‑scale gold mining contribute to marine mercury contamination. Communities far from mining sites are at risk because mercury bioaccumulates in coastal food webs (see supplement AB Keshk).

### Informal recycling and e‑waste

The formal and the informal recycling sectors frequently function in a manner that is not subject to the constraints of regulatory frameworks. These sectors are considered to be significant contributors to exposure to toxic metals, a phenomenon that is particularly evident in LMICs, where especially the infrastructure for the formal waste disposal system is considered to be inadequate [5].

Abigail Serwaa Akoto Bawua (University of Ghana) presented on the Agbogbloshie dumpsite in Accra, Ghana, which is one of the world’s largest informal e‑waste recycling hubs. Informal workers, including children, dismantle discarded electronics using methods such as open burning and acid baths to extract valuable metals. These methods release lead, copper, cadmium, arsenic, and flame retardants into the environment. A cross‑sectional study involving 56 pupils from 4 schools located within a 1‑km radius of the e‑waste site revealed elevated levels of toxic metals, with mean blood lead levels (BLLs) at 60.4 µg/L and urinary arsenic levels at 21.50 µg/L [18]. Earlier studies documented higher BLLs in Agbogbloshie. According to the findings of Puschel et al. [19], 77.7% of the participants had BLLs that exceeded the 50 µg/L guidance level established by the WHO. CDC and WHO state that there is no known safe blood lead concentration in children and adults. According to WHO, “the source(s) of lead exposure should be identified, and appropriate action taken to reduce and terminate exposure” [20]. According to Njoku et al. [21], Africa has been the site of e‑waste disposal from the developed world, resulting in the establishment of a substantial informal e‑waste recycling sector in Ghana.

Andrea Kaifie‑Pechmann (FAU Erlangen) presented that over 80% of e‑waste workers had exposure levels of carcinogenic inorganic arsenic species that exceeded acceptable concentrations (see supplement AB Kaifie‑Pechmann). Johanna Elbel (Sciences Po Paris) presented on socio‑ecological fieldwork. Despite the demolition of the Agbogbloshie scrapyard in 2021, workers resumed burning operations nearby and returned to the scrapyard four years later. This highlights resistance to coercive, top‑down approaches, the persistence of informal economies, and the shortcomings of enforcement‑only strategies (see supplement AB Elbel).

Similar patterns can be observed in Latin America and Asia, where people use backyard workshops to disassemble e‑waste. Dewi Yunia Fitriani (Universitas Indonesia) explained how the informal recycling of ULABs exposed peri‑urban areas around Java Island to lead and cadmium. Since families share living and working spaces, it is difficult to distinguish between occupational and environmental exposures, respectively (see supplement AB Fitriani). This was evidenced in a presentation by John Yabe (University of Namibia), who described alarming environmental lead exposure in children near a lead‑zinc mine in Kabwe town, Zambia. John Yabe also described recent reports of elevated BLL in children in a similar setting in Namibia. Toddlers, who ingest dust through hand‑to‑mouth activity, are particularly vulnerable. Even large‑scale facilities are not immune (see supplement AB Yabe). Andreas Manhart and Anuradha Varanasi (both Oeko‑Institut) reported that registered ULAB recycling plants in Africa and India often operate below international safety and emissions standards, releasing substantial lead into workplaces and surrounding communities (see supplement AB Manhart). These findings demonstrate that, without adequate regulation, technology, and worker protection, recycling, which is one of the components of the circular economy, can perpetuate toxic exposures. Formal lead‑recycling factories in Ogijo, Nigeria, send recycled lead for US car batteries while spewing toxic dust that poisons workers and residents, especially children. Soil and blood tests show dangerously high lead levels, highlighting regulatory failure and global supply‑chain impacts.^4^

In 2007 and 2008, a total of 18 children died from lead (Pb) poisoning in Dakar, Senegal. The poisoning was caused by the children inhaling and ingesting soil and dust with high lead contamination that was attributed to unsafe ULAB recycling practices [22]. This case study elucidates the risks associated with the recycling of lead‑acid batteries.

### Legacy contamination and persistent urban sources

Although modern mining and recycling receive attention, legacy contamination remains a significant source of chronic exposure. Decades of leaded gasoline use, historical smelting sites, and abandoned mines have left millions living in neighborhoods where soil lead, cadmium, and arsenic levels exceed safe thresholds.

Kabwe mine town, which is ranked among the most polluted places on Earth, serves as a practical example of legacy environmental exposure arising from historical lead‑zinc mining activities in Zambia, which operated from about 1904 to 1994.

Already in 1975, A. R. L. Clark wrote his thesis on lead poisoning in Kabwe, documenting severe childhood lead exposure in Kabwe due to emissions and waste from lead mining and smelting. Children showed high BLLs, with cases of lead encephalopathy, seizures, coma, and death in affected neighborhoods. Elevated cord blood lead confirmed prenatal exposure, while reduced birth weight, altered blood parameters, and bone “lead lines” showed chronic toxicity. Exposure was involuntary, continuous, and preventable. Clark concluded that basic environmental controls, screening, and treatment could have averted harm and that Kabwe’s children functioned as unprotected bystanders to industrial production [23].

John Yabe (University of Namibia) highlighted the impact of lead contamination on the environment, animals, and humans in a one health approach (see supplement AB Yabe). Comprehensive studies in Kabwe revealed alarming BLLs in children, ranging from 33 to 1270 µg/L, meaning that almost 100% of the children exceeded the 35 µg/L Centers for Disease Control and Prevention (CDC) reference level of concern [24, 25].

Lea John (LMU Munich) also presented alarming results from the BLENCA2 study conducted in Goslar, Germany. In this study, 51% of the children screened had BLLs that exceeded the German reference values; 24% surpassed the CDC threshold of 35 µg/L; and 13% exceeded the WHO guidance level of 50 µg/L (see supplement AB John). These data demonstrate that the Goslar district of Germany, historically used for intensive mining activities resulting in long‑term environmental contamination, particularly with lead, still experiences exposure today due to this legacy [26].

Beyond mining legacies, other activities pose ongoing risks. Melissa McDiarmid and Joanna Gaitens (both University of Maryland) reported on war‑exposed military populations in whom depleted uranium fragments embedded in soft tissue continued to release uranium decades after exposure. Biomonitoring confirmed the persistence of uranium 30 years after exposure [27].

Seasonal and cultural practices can also create acute pulses of exposure. Tushar Joshi (Maulana Azad Medical College) found that metal concentrations in ambient air and urine spike significantly during Diwali due to firecracker combustion, with elevated levels of lead, copper, and strontium above baseline. These episodic surges add to the chronic burden of urban exposures (see supplement AB Joshi).

Katharina Deering and Stefan Rakete (both LMU Munich) showed that toxic metals such as arsenic, mercury, and lead have been used historically as preventive and curative conservation agents for cultural and biological museum collections to protect them from pests and mold [28]. However, these substances pose health risks to staff handling contaminated objects. The results of the study show that arsenic, lead, and mercury accumulate in household and museum dust, correlating with proximity to industrial sites and major roads.

### Agricultural activities and consumer products

Even beyond extraction and recycling, modern industrial activities like agriculture continue to introduce toxic metals into ecosystems. For example, applying phosphate fertilizers can significantly increase the concentration of potentially dangerous trace elements, such as lead, cadmium, and arsenic, in the soils of croplands. These elements can move up the food chain and accumulate in soil [29]. Employees in the leather and electroplating sectors may come into contact with dangerous materials such as hexavalent chromium (chromium VI and chromium trioxide) and nickel compounds (such as nickel sulfate and chloride). Humans are frequently exposed to the metalloid arsenic in food, water, air, and soil. Syarifah Hidayah Fatriah (Universitas Muhammadiyah Riau, Indonesia) reported that arsenic has a long history of use as a homicidal agent but has also been used as a pesticide, chemotherapeutic agent, and ingredient in consumer goods during the last century (see supplement AB Fatriah). High levels of naturally occurring arsenic in drinking water are a toxicological concern in some parts of the world. As discussed during the policy panel, these diffuse sources often lack robust monitoring, which allows for cumulative exposures that compound the risks of point‑source contamination.

Jenna Forsyth (Stanford University) presented evidence of widespread adulteration of spices with lead chromate throughout India. Expanding upon past efforts [30], her team collected over 4000 samples of spices, including turmeric, chili, cumin, coriander, and mango powder, from 104 major cities. Their findings showed that 7% of turmeric samples exceeded the national limit of 10 mg/kg for lead and contained elevated chromium, suggestive of adulteration with lead chromate pigments. Some samples had lead levels over 8000 mg/kg, posing severe health risks. Notably, 90% of the lead‑containing samples were turmeric roots, suggesting that lead chromate adulteration and contamination occurred early in the supply chain. Prior studies have determined that lead chromate is added to spices primarily to increase their visual appeal and color but may also increase shelf life [31]. Forsyth called for immediate action to stop the use of toxic additives in the spice trade, citing successful examples from Bangladesh [32] and Georgia [33] where improved awareness, detection, and regulatory enforcement dramatically reduced spice lead levels.

Adna Alilović (Jožef Stefan Institute) presented new evidence on mercury exposure via tuna consumption, focusing on controlled dietary intake and biomarker responses in humans. Fish provides important nutrients but is also the main source of methylmercury (MeHg) exposure in humans. This study examined mercury and selenium kinetics during controlled tuna consumption. Ten participants ate five tuna steaks over five days while six controls avoided seafood. MeHg made up 84% of total mercury and selenium levels in tuna correlated with mercury. Blood mercury rose linearly and declined biphasically, while urinary mercury peaked later. Selenium biomarkers showed compartment‑specific patterns. Findings indicate complex, time‑dependent Se–Hg interactions that remain insufficiently understood and warrant further research (see supplement AB Alilović).

Martí Nogués Freixas (IDAEA‑CSIC, Barcelona) presented data on mercury exposure linked to dietary patterns in Northern and Southern Europe. Hair biomonitoring from adolescents in Poland and adults in Barcelona revealed substantially higher mercury levels in Mediterranean populations, likely driven by higher fish consumption. The findings indicate that diet strongly influences internal mercury burden and that larger cohorts and refined dietary assessments are needed to confirm these exposure pathways (see supplement AB Freixas).

Konstantinos C. Makris (Cyprus University of Technology) presented findings from a cluster‑randomized crossover trial assessing whether an organic food intervention could reduce children’s exposure to lead and cadmium. In 149 primary school children, the 40‑day organic food period reduced urinary lead levels over time, with oxidative damage biomarkers correlating positively with metal biomarkers. The results suggest that dietary interventions may lower metal body burden, underscoring the need for additional human studies in high‑exposure settings (see supplement AB Makris).

Aelita Sargsyan (Pure Earth) identified lead in consumer products as the primary source of exposure, focusing on market goods identified through rapid screening methods. They screened 5007 items across 25 LMICs: 51% of metal foodware, 45% of ceramic foodware, and 41% of paints exceeded international thresholds. Some cosmetics such as kohl contained >600,000 mg/kg lead. As of January 2024, 48% of countries had legally binding controls limiting lead in new paints, often adopting the 90 mg/kg standard. However, the study by Sargsyan et al. found that in several countries and Indian states with such regulations, including Colombia, Kenya, Mexico, Pakistan, and others, over 10% of sampled paints still exceeded 90 mg/kg. These findings indicate significant gaps in enforcement and compliance, suggesting that regulatory measures alone are insufficient without effective monitoring and implementation mechanisms [34].

Rachel Bonnifield (Center for Global Development) highlighted how contaminated consumer goods, including spices and toys, have undermined child health interventions in LMICs (see supplement AB Bonnifield). Marcial Velasco‑Garrido (Universitätsklinikum Hamburg‑Eppendorf) presented a case study documenting Ayurvedic pharmaceuticals containing enough lead to raise blood levels above 700 µg/L (see supplement AB Velasco‑Garrido).

Andrea Hartwig (Karlsruhe Institute of Technology, KIT) presented an overview of the toxicology of aluminum based on extensive literature evaluated by the German MAK Commission and discussed different threshold values for aluminum compounds that have been established. She highlighted that although dermal and oral absorption are low, inhalation, particularly in occupational settings and via aerosolized antiperspirants, represents a relevant exposure route associated with neurotoxicity, irritation, and inflammatory responses. Current evidence does not support a causal link with breast cancer (see supplement AB Hartwig).

Samuel Tetsopgang (INTEV, Cameroon) presented an estimation of mercury releases from broken medical thermometers discarded in Cameroonian hospitals. Survey data indicated widespread use and breakage of mercury thermometers, leading to substantial local emissions. Although small in global terms, these releases are locally significant and comparable to a notable share of ASGM‑related mercury use (see supplement AB Tetsopgang).

## Health Hazards of Toxic Metals

### Neurodevelopmental effects

The neurotoxicity of lead, mercury, manganese, and other metals remains a cornerstone of public health concern. Even low‑level exposure in early life is strongly linked to reduced IQ, behavioral problems, and attention disorders [35–38]. The symposium highlighted a relationship between neurodevelopmental risks and environmental exposure to some toxic metals, particularly among children living in polluted environments.

Dewi Yunia Fitriani (Universitas Indonesia) presented new data showing average BLLs of 80–120 µg/L among preschool children in urban slum areas of Jakarta, correlating with a 3–5 point IQ deficit compared to peers in low‑exposure districts (see supplement AB Fitriani). In addition, John Yabe (University of Namibia) presented concerns of neurodevelopmental impairment in Kabwe, Zambia (see supplement AB Yabe). Using the Ages‑Stages (ASQ‑3) screening tool, lower neurodevelopmental scores, including personal social, problem‑solving, fine motor, gross motor, and communication, were associated with high BLLs among children living in the vicinity of a former lead‑zinc mine compared with children from communities further away from the mine, in the same town. A presentation by Abigail Serwaa Akoto Bawua (University of Ghana) reported cognitive dysfunction symptoms in children from schools near Accra’s general scrapyard Agbogbloshie, which is an e‑waste dumpsite in Ghana, where 75% of children experienced confusion, 67.9% had poor memory, and 66% had difficulty concentrating [18]. Elevated levels of toxic metals, such as lead (mean BLLs of 60.4 µg/L) and arsenic (mean urinary arsenic of 21.50 µg/L), were measured in the children [18].

Mercury exposure, primarily from ASGM and contaminated fish, impairs fetal and child brain development [39]. Stephan Bose‑O’Reilly (LMU Munich) reported that prenatal methylmercury exposure is associated with deficits in language, memory, and motor function, effects that are irreversible and persist into adulthood, as recently addressed in critical reviews of the literature [40, 41]. Adna Alilovic (Jožef Stefan Institute) added evidence from Eastern Europe showing similar neurocognitive impacts among children in mercury legacy hotspots (see supplement AB Alilovic).

### Carcinogenicity and organ toxicity

Metals such as arsenic, cadmium, nickel, and chromium are classified by the International Agency for Research on Cancer (IARC) as Group 1 carcinogenic to humans. Kurt Straif (Boston College) synthesized recent epidemiological findings linking occupational exposures to these metals to lung, skin, and bladder cancers, especially in metal plating and mining sectors (see supplement AB Straif). Ibrahim Issah and Julius Fobil (University of Ghana) presented a systematic review showing elevated liver enzyme biomarkers and genotoxic damage among communities exposed to mixed metal pollution in Ghanaian mining towns [42]. In a study around the Agbogbloshie scrapyard in Ghana, exposed individuals had BLLs >100 µg/L and some individuals presented renal disorders consistent with high lead exposure [19]. Evidence of hepatic and renal toxicity was reported by John Yabe (University of Namibia) in populations living near a lead‑zinc mine in Kabwe, Zambia, where 36% and 42% of the study participants exhibited elevated lactate dehydrogenase (LDH) and gamma‑glutamyl transferase (GGT), respectively. Moreover, creatinine levels were elevated in 34% of the participants (see supplement AB Yabe).

Dragana Vukelić (University of Belgrade) presented benchmark dose modeling data on lead toxicity in Wistar rats to identify sensitive toxicological endpoints. Chronic low‑dose exposure produced dose‑dependent effects, with testosterone reduction emerging as the most sensitive endpoint, followed by alterations in oxidative stress markers and bioelements. These findings provide mechanistic insights and support reference point development for human health risk assessment (see supplement AB Vukelić).

Chronic cadmium exposure, often via diet and smoking, is associated with kidney dysfunction, osteoporosis, and increased cancer risk. Combined exposure, such as co‑exposure to cadmium and arsenic in rice‑growing regions, presents complex risk interactions that are still under‑researched but likely amplify harm. For example, combined exposure to cadmium and inorganic arsenic was related to considerably more elevated levels of indicators of kidney dysfunction in a metal‑exposed population group than in groups exposed only to one of these agents [43].

Evidence on health effects for some of the critical raw materials needed in the green transition is more limited. In a Swedish study, recyclers (of among others e‑waste) had high concentrations of 14 different metals in blood and/or post‑shift urine compared to controls [4]. One of these metals was indium where post‑shift urinary concentrations were up to 3 µg/L in urine of exposed workers (median 0.11) versus a median concentration of 0.05 and 0.03 µg/L among low‑exposed and control groups [4]. Indium is known for its long half‑life in serum after exposure to poorly soluble indium compounds (up to nine years) due to its accumulation in the lungs [44]. Indium tin oxide is classified as possibly carcinogenic to humans (2B), and in occupational settings, indium is known to cause interstitial lung disease, emphysema, pulmonary alveolar proteinosis, and lung cancer [44].

Uranium mining poses serious environmental and health risks due to exposure to ionizing radiation and the release of radon gas. It could increase in the coming years if construction of new nuclear reactors moves forward in response to the false claim that nuclear energy represents a “clean, reliable, safe, and affordable” alternative to fossil fuels. Uranium miners face an increased risk of lung cancer due to their exposure to radon [11], which is magnified by cigarette smoking [12], while nearby communities may experience kidney damage from contaminated groundwater [45]. Nuclear waste from reactors remains radioactive for millennia and poses grave hazards to nuclear workers, transport workers, and residents of communities where this waste is stored. In addition, there is the perennial possibility that nuclear materials may be diverted to weapons production by malign actors. These impacts underscore the need for stricter regulation and monitoring of uranium extraction and waste management practices.

### Cardiovascular and metabolic effects

At the symposium, the issue of toxic metals exposure and their effects on the cardiovascular system was raised. Emerging evidence links low‑level lead exposure to hypertension, atherosclerosis, and increased risk of stroke and heart disease [46–48]. At the symposium, Björn Larsen and Ernesto Sanchez‑Triana (both World Bank) highlighted the significant cardiovascular disease (CVD) burden linked to lead exposure, particularly in LMICs. They emphasized that lead‑induced hypertension and heart disease contribute substantially to global mortality, often exceeding the burden from other well‑known environmental risk factors [49]. Also, Björn Larsen pointed out that mercury exposure contributes substantially to the global burden of disease, both due to the neurotoxic prenatal effects (already mentioned in section “Neurodevelopmental Effects”) and the cardiovascular effects in adults. Such effects in adults were, for example, reported in a population with elevated blood and hair mercury in combination with low levels of polyunsaturated fatty acids [44]. Björn Larsen and Ernesto Sanchez‑Triana called for stronger global policy actions and preventive measures to reduce lead and mercury exposure and their far‑reaching health and economic impacts.

### Reproductive and developmental toxicity

Some metals, including lead, mercury, nickel, cobalt, and certain chromium compounds, have been implicated in reproductive toxicity, affecting fertility and increasing the risk of adverse pregnancy outcomes. In mining communities, high maternal body burdens correlate with premature births and low birth weight [50]. Also, as mentioned above, methylmercury and lead affect children’s neurodevelopment negatively.

Paul Musa Obadia (University of Lubumbashi) highlighted the reproductive effects of metals in male miners in the Democratic Republic of Congo [9]. Smelter workers showed high blood cobalt (0.82 µg/L) and lead (190 µg/L), and elevated urinary cobalt (10.7 µg/g creatinine), germanium (0.15 µg/L), and lead (9.51 µg/L). The miners had lower International Index of Erectile Function (IIEF) scores (66 vs. 73), lower free testosterone (8.11 vs. 10.52 ng/dL), and greater odds of erectile dysfunction (aOR, 2.6; 95% CI, 1.3–5.3) compared to non‑exposed males [9] (see supplement AB Musa Obadia).

Christine Rösch (CFCS‑Consult GmbH) presented challenges of genotoxicity testing for metals under the REACH framework. She emphasized that standard in vitro assays, optimized for organic chemicals, often yield misleading or non‑predictive results for metals due to differences in absorption, distribution, metabolism, and excretion (ADME) processes, transport mechanisms, and indirect effects. Tailored testing strategies are therefore essential to avoid artifacts and ensure meaningful regulatory outcomes (see supplement AB Rösch).

### Vulnerable populations and inequity

In a comprehensive study involving over 10,000 children in Kabwe, Zambia, Tiza Mufune (CiH LMU, Ministry of Health Zambia) described an association between elevated BLLs and anemia, where the mean Hb was 12.3 g/dL, with the prevalence of anemia being 23.4% (see supplement AB Mufune).

Krishna Nirmalya Sen (Larsen & Toubro Limited, India) presented strategies for protecting workers from toxic metal exposures during brownfield expansion projects in the minerals and metals sector. He emphasized that such projects require proactive OHS frameworks aligned with “Vision Zero,” combining safe processes, competent workforces, and digital technologies. This integrated approach improved safety performance, reduced incidents, and demonstrated that leadership commitment and multipronged risk management are essential for future modernization efforts (see supplement AB Sen).

Health is affected in multiple ways (see Table 1). A recurring theme from symposium presenters was the disproportionate burden borne by marginalized groups, including the informal miners, recyclers, and low‑income urban communities. Children, pregnant women, and workers in informal sectors face the highest risks yet often lack access to healthcare and protective regulations. This reality underscores the need for targeted interventions and equity‑focused policies alongside technological solutions.

**Table 1:** Summary of key toxic metals, main sources, and primary health effects.

METAL	MAIN SOURCES	PRIMARY HEALTH EFFECTS
Lead (Pb)	Recycling of used lead‑acid batteries, legacy mining, contaminated soil and paint, small aircraft fuel, commodity products	Cardiovascular mortality, Neurodevelopmental impairment, kidney disease
Mercury (Hg)	Artisanal and small‑scale gold mining (ASGM), fish consumption, industrial emissions, coal burning	Cognitive deficits, kidney toxicity, cardiovascular disease, neurological symptoms
Arsenic (As)	Drinking water, mining and smelting, e‑waste	Cancers of the skin, bladder and lung, skin lesions other than skin cancer, spontaneous abortion, stillbirth, infant mortality, congenital heart disease, respiratory disease, chronic kidney disease, neurodevelopmental effects, ischemic heart disease and carotid artery atherosclerosis
Cadmium (Cd)	Mining, battery recycling, fertilizers	Kidney dysfunction, osteoporosis, cancer, cardiovascular disease
Uranium (U)	Mining, processing, military	Kidney dysfunction, cancer
Chromium (Cr)	E‑waste, tanning, metal plating	Neurodevelopmental issues, stomach cancer
Nickel (Ni)	Battery recycling, mining, refining	Dermatitis, occupational asthma, cancer
Cobalt (Co)	Battery recycling, mining, refining	Dermatitis, occupational asthma, cancer
Lithium (Li)	Battery recycling, mining, refining	Irritation of airways and skin, reproductive effects

Sources: References [49, 51–54].

## Advances in Monitoring and Risk Assessment, Gaps

### Advances in monitoring

Effective monitoring and risk assessment are central to control toxic metal exposure in communities and workplaces. Over the past decade, technological progress and community‑driven innovations have made toxic metal analysis in environmental and biological samples more affordable and actionable. However, exposure assessment remains inconsistent, particularly in LMICs where surveillance capacity and laboratory infrastructure are often limited for manifold reasons, for example, lack of funds for equipment and personnel, no or unreliable access to electricity, maintenance, and consumables, as well as restricted accessibility to the areas and people of interest.

At the *Toxic Metals Symposium 2025*, multiple presenters showcased scalable techniques to fill these gaps. Stefan Rakete (LMU Munich) informed about the potential and current limitations of microsampling‑assisted biomonitoring methods for toxic metals in blood (see supplement AB Rakete). Microsampling uses minimal capillary blood volumes, usually less than 100 µL. It does not require venous blood sampling or cooling chains and can simply be shipped by mail [55]. Thus, microsampling complements or supports exposure assessment in areas where conventional blood sampling methods are difficult to conduct, for example, in ASM areas. These minimally invasive methods will become critical for screening vulnerable populations, especially children in remote areas where clinical facilities are scarce. Although there is a growing amount of research on toxic metal analysis in blood using microsampling, only a few studies actually applied microsampling, mostly dried blood spots (DBS), in field studies [55–59]. Today, the focus shifts from DBS to novel devices such as volumetric absorptive microsampling (VAMS) or quantitative DBS (qDBS). However, its applicability in field studies remains to be seen as multielement microsampling methods have thus far been developed for laboratory or near‑laboratory conditions [60–62].

Don Smith (University of California) described advances in analytical methods to inform environmental exposure assessment that included advanced stable isotope mass spectrometry and laser ablation mass spectrometry methods (see supplement AB Smith).

Community biomonitoring emerged as a practical complement to technological advances. Yi Lu (Vital Strategies) presented on monitoring lead exposure in Indian children. Her work emphasized the importance of systematic surveillance to assess BLLs in vulnerable populations. She advocated for government‑led monitoring efforts to better understand exposure patterns and to support targeted interventions and policy responses that protect child health [63, 64]. Tiza Mufune (CiH LMU, Ministry of Health, Zambia) reported on the correlation between lead exposure and childhood anemia and stunting in Zambia, stressing the need for improved monitoring and health data integration to inform public health action (see supplement AB Mufune). Abigail Akoto Bawua (University of Ghana) presented a hybrid model combining school‑based lead testing with parental engagement workshops, showing how awareness can increase demand for municipal interventions [18].

Katharina Deering (LMU Munich) demonstrated the importance of indoor exposure assessment through low‑cost passive dust traps. Her German case study revealed that while ambient air pollution has declined in Europe, indoor metal reservoirs, legacy lead paint, and contaminated carpets still contribute substantially to children’s exposure [28]. Don Smith (University of California) emphasized that risk assessments should therefore combine external environmental data with biomarkers (blood, urine, hair, etc.) for a complete picture (see supplement AB Smith).

Quality control (QC) procedures verify that analytical measurements of toxic metals are reliable during routine operation. Effective QC detects contamination, instrument drift, interferences, and operator errors that could distort concentration estimates. Because health and regulatory decisions depend on accurate exposure data, QC is essential for credible risk assessment. This is especially critical in LMICs, where laboratory infrastructure may be limited, and measurement uncertainty can otherwise compromise public health interventions, environmental monitoring, and policy responses to toxic metal hazards [29, 65].

### Risk assessment

The symposium explicitly highlighted risk assessment, alongside environmental and human biomonitoring, as a core topic aimed at evaluating and mitigating toxic metal exposures.

Healthcare strategies should be tailored to the specific exposure context, including occupationally exposed workers and the general population. Within the latter, age groups need to be distinguished, and sensitive subpopulations identified, as children, pregnant women, and the elderly may exhibit higher toxicokinetic susceptibility to metal exposure. Recognizing these differential vulnerabilities is essential for risk assessment and for designing preventive, diagnostic, and treatment measures that adequately protect all exposed groups [66].

The symposium emphasized the need to assess health risks from low‑level exposure to metals like lead, mercury, arsenic, and cadmium, as well as emerging metals used in new technologies and their links to CVD, cancer, and cognitive disorders. Together, these points reflect the symposium’s focus on establishing strong frameworks for evaluating exposure risks and guiding policy interventions.

### Gaps in monitoring

Despite progress, challenges persist. Stefan Rakete (LMU Munich) and Yi Lu (Vital Strategies) highlighted the scarcity of reference laboratories in LMICs capable of confirming field results (see supplement AB Rakete and AB Lu Y). Moreover, cross‑border data comparability is hindered by inconsistent sampling protocols and reporting units. Several symposium participants advocated for a global framework, analogous to the WHO’s Global Mercury Observation System, but expanded to cover multi‑metal surveillance with clear quality standards. There are also considerable knowledge gaps concerning the health effects of mixed exposures, exposure to rare‑earth elements as well as nanoparticles both in the general population and in occupational settings.

Beyond this, translating data into action is critical. Some countries lack robust health risk assessment capacity or trained toxicologists to interpret findings. Don Smith (University of California) noted that rapid data collection without local capacity can cause community alarm when there are no resources for remediation. Effective risk communication must therefore accompany monitoring, ensuring that affected communities understand results and options for exposure reduction.

In summary, advances in detection technology and grassroots monitoring hold promise for bridging exposure data gaps. However, scaling up these tools requires international investment, capacity building, and integration into national environmental health systems.

The symposium consensus was clear: without sustained monitoring, toxic metal hazards remain invisible and unaddressed.

## Policy and Governance Challenges

Despite decades of scientific evidence on the health impacts of toxic metals, policy responses remain uneven and often reactive. The Toxic Metals Symposium 2025 highlighted persistent governance challenges that undermine efforts to mitigate exposure risks, particularly in LMICs supplying the minerals that are essential for sustainable resource efficiency within the green transition [67].

Policy and governance challenges relate to policy design and implementation (that includes funding and enforcement). Policy failures are often impacted by the lack of inclusion of essential stakeholders in the policy design process, and governance issues can result from, first, the lack of recognition of the needs of these groups, and second, short‑term political and economic visions.

Unwrapping these challenges, it is important in policy development to move beyond shortsighted evaluation and to include downstream assessments of policy impacts as well as recognition of informal workers’ needs. Rachel Bonnifield (Center for Global Development) discussed how well‑intentioned development aid projects can inadvertently exacerbate lead hazards. She presented a case where road construction and urban upgrading in West Africa displaced informal recyclers into new settlements without waste management infrastructure, creating uncontrolled dumping grounds for batteries and electronics (see supplement AB Bonnifield).

Informal sector workers pose a unique governance dilemma, as the sector makes up a large part of local economies (up to 90% in SSA countries) and societies have been structured around these informal sectors for centuries [68]. Criminalizing these activities without providing safer formal alternatives perpetuates poverty and hazardous practices [69, 70]. Similar to conclusions drawn from Rachel Bonnifield (Center for Global Development), Johanna Elbel (Sciences Po Paris) showed that coercive policy approaches to control the informal workers in Ghana fail as workers show resistance (see supplement AB Elbel). Several presenters, including Laura Emilce Flores Rodriguez (Hospital de Clínicas Paraguay) and Andrea Kaifie‑Pechmann (FAU Erlangen), called for inclusive policies that recognize informal workers as stakeholders, providing them with training, protective equipment, and pathways to legalization (see supplement AB Flores and AB Kaifie‑Pechmann).

Efforts to overcome shortsighted policies and shift toward sustainable pose additional challenges. At the national level, long‑term political will and sustainable financing of initiatives are necessary but are often lacking in donor‑dependent contexts. Pamela Mwanza (Zambia Environmental Management Agency) offered insight into regulatory enforcement challenges in resource‑rich LMICs. Zambia has implemented lead abatement programs in Kabwe, but struggles, like other SSA countries, with sustained funding and political continuity. A change in local leadership often derails clean‑up budgets and staff retention.

High levels of corruption and lack of transparency further erode trust, impede environmental regulation, and diminish the sustainability of programs. Furthermore, community representatives at the symposium recounted cases where mining companies evaded penalties through political connections, leaving residents to bear the health burden.

The increasing operational complexity of transnational extractive companies and the absence in many jurisdictions of enforceable legal standards on environmental harm and human rights result in many transnational corporations acting with impunity. There are very significant obstacles to individuals and communities gaining access to remedies where toxic metals contamination has occurred. Sophie Turner (Leigh Day, UK) emphasized the importance of due diligence regulation, noting that transnational corporations often benefit from weak local governance and low fines for non‑compliance with local standards and regulations. She argued for binding and enforceable due diligence laws requiring companies to monitor and report environmental harm such as toxic metals contamination throughout their supply chains. Such a framework now exists in the EU in the form of the Corporate Sustainability Due Diligence Directive (CSDDD) but remains absent in many jurisdictions.

In the absence of governmental regulation, strategic civil litigation can help to fill the gap and has emerged as a vital mechanism for advancing corporate accountability and offering a route for victims to access justice, through the advancement of legal claims against parent companies in their home jurisdictions for harm caused by their subsidiaries overseas. Recent years have seen a significant proliferation of such litigation, particularly in England, where the courts have been at the forefront of developing this novel field of corporate accountability law. When successful, such legal claims can result in damages, remediation, and restorative measures that can help individuals and communities adversely affected by toxic metal contamination to rebuild their lives.

Acceptable exposure levels for lead, cadmium, and mercury differ significantly across countries, complicating multinational supply chain regulation. Don Smith (University of California) and other speakers stressed that WHO guidelines should serve as a global minimum standard, with countries encouraged to adopt stricter national standards based on local vulnerability and capacity.

Complementing comments made on international regulations and Corporate Responsibility (CR), symposium participants reiterated the lack of international harmonized standards and guidance. Acceptable exposure levels for lead, cadmium, and mercury differ significantly across countries, complicating multinational supply chain regulation. Don Smith (University of California) and other speakers stressed that WHO guidelines should serve as a global minimum standard, with countries encouraged to adopt stricter national standards based on local vulnerability and capacity.

Taken together, these policy failures and challenges at the community, national, and international levels highlight the need for stronger international cooperation and guidance, sustainably funded regulatory bodies, community recognition and participation in decision‑making, and corporate accountability mechanisms on metals. Only through such multilevel governance and multistakeholder engagement can countries prevent the green transition from entrenching environmental injustices. Such sustainably funded, international cooperation on metals will protect human and planetary health and advance social justice. They will comply with the 2025 ruling by the International Court of Justice that all UN Member States are bound by a collective responsibility to collaborate to ensure the preservation of the Earth’s environment.^5^

## Roadmap for a Safe and Equitable Green Transition

Based on the findings of the Toxic Metals Symposium held in July 2025 in Munich, this roadmap outlines strategies countries can deploy to advance a green transition that protects people and ecosystems from the growing risks posed by toxic metals. The symposium made clear that while metals are indispensable for the energy transition, their use must be judiciously managed. Past industrial expansions have shown the consequences of overlooking health risks. Repeating those mistakes of the past will undermine both public trust and climate goals. Policymakers therefore face a dual imperative: to accelerate decarbonization while ensuring that new technologies do not introduce fresh hazards or amplify existing inequalities. The Toxic Metals Symposium 2025 highlighted the urgent need to set priorities by estimating the benefits and costs associated with those chemicals, and to design and implement policies to address the priority toxic chemicals.

Setting priorities for toxic chemicals is crucial because the number of substances in commerce and the scale of pollution far exceed the capacity of scientific and regulatory systems to assess and manage all risks at once. Impact‑focused prioritization helps ensure that limited resources are directed toward chemicals that cause the greatest harm to human health, ecosystems, and socioeconomic systems, rather than being dispersed across lower‑impact or poorly defined issues. Evidence from impact‑based frameworks and expert‑judgment studies shows that a small subset of chemicals is responsible for a disproportionate share of global disease and mortality, particularly in LMICs where exposure levels are high, and data gaps persist.

By focusing on chemicals with the largest and best‑documented impacts, priority setting can improve the efficiency, effectiveness, and equity of environmental health policies. Setting priorities allows policymakers and researchers to align actions with interventions that yield the greatest public health benefits. Within this context, priority toxic metals such as mercury, lead, and cadmium, which have been conclusively shown to impair cognitive development in children and to be significant risk factors for CVD and premature mortality in adults, underscore the importance of targeting these substances and considering their removal from the global economy [71, 53].

A first priority is to establish robust systems for monitoring exposures and health outcomes, particularly among workers in mining, processing, recycling, and other high‑risk sectors of the metals industry. Without reliable surveillance, governments cannot design effective protection strategies or identify emerging threats. Policymakers should expand community‑based monitoring and invest in accessible biomonitoring tools, enabling rapid detection of hotspots and quicker public‑health responses. Across all regulatory decisions, the precautionary principle should guide action, recognizing that many newer metals and compounds still lack comprehensive toxicological evidence.

As countries scale up green industries, greater international coordination will be essential. Multilateral governance is especially important because the supply‑demand chains for critical metals cut across borders, and exposures in one country often stem from consumption patterns in another. Harmonizing health‑based standards for well‑studied metals such as lead, mercury, and cadmium, using WHO guidelines as global minimum standards, is an achievable early step, especially in the light of current geopolitical shifts. For emerging metals, including lithium, cobalt, and rare earths, policymakers should work toward jointly developed, regionally relevant benchmarks that can evolve as scientific evidence progresses.

To reduce emissions at source, governments should promote cleaner technologies in mining, smelting, and recycling, offering incentives for best available techniques while enforcing stronger penalties for unsafe practices. Supporting the informal sector is also vital. Many artisanal miners and recyclers work under hazardous conditions driven by poverty. Recognizing their labor, providing training and protective equipment, and integrating them into formal regulatory systems can significantly reduce exposures. Technology can further strengthen monitoring: investments in digital records, electronic reporting, and artificial intelligence can improve transparency, support compliance, and inform regulatory planning.

Over the longer term, structural reforms and adequate financial mechanisms will be required to ensure lasting protection. Governments should adopt due‑diligence legislation obliging companies to identify, prevent, and remediate toxic metal risks throughout their supply chains. One approach may be to require companies to pay in advance into contingency funds that will be available for environmental remediation. Similarly, climate finance mechanisms should explicitly include funding for toxic‑metal monitoring and cleanup, ensuring that the costs of pollution are not externalized to vulnerable communities. Policymakers should also support research on cumulative and intergenerational effects, safer substitutes for hazardous inputs, and scalable remediation approaches. Establishing reference guidelines, especially where national standards are absent, would help align national policies with global good practice.

Nuclear power should not be part of the green transition because it introduces serious health hazards. Uranium, radon, and other radioactive by‑products pose long‑term risks to workers and nearby communities, and managing radioactive waste remains an unresolved danger. Despite claims that nuclear energy is a safe and affordable alternative, it is costly, slow to build, and unnecessary given the growing capacity of renewables. Policymakers should avoid creating new toxic exposures and exclude nuclear power from future energy strategies.

Several countries may choose to create dedicated governmental entities to coordinate toxic‑metal policy, ensuring consistent oversight and linking occupational health, environmental protection, and industrial development. In the international arena, a United Nations political declaration on toxic metals could mobilize political commitment, set shared goals, and facilitate cooperation across regions.

Across all phases of metal mining, processing and recycling, community engagement, social equity, and respect for Indigenous rights must remain central. Those most affected by exposure often have the least influence over policy decisions. Policymakers therefore have a responsibility to involve affected communities in decision‑making, strengthen local capacity, and ensure that the benefits and burdens of the green transition are distributed fairly.

Taken together, this roadmap (see Table 2) translates the symposium’s scientific insights into practical measures that can guide countries toward a green transition that is not only climate‑ambitious but also safe, equitable, and protective of human health.

**Table 2:** Roadmap for mitigating toxic metal hazards.

LEVEL	KEY ACTIONS	STAKEHOLDERS
**Foundational Prevention**	Systematically monitor exposures and health outcomes; apply the precautionary principle; strengthen surveillance in high‑risk sectors	National governments, work environment, public health and environment agencies, research institutions, industry
**Sustainable Production and Regulation**	Protect workers by promoting cleaner mining, smelting, and recycling technologies; harmonizing health‑based standards; formalizing informal workers; strengthening enforcement	Governments, industry, labor organizations, NGOs
**Equitable Community Protection**	Protect communities by expanding community engagement; ensuring social protection for vulnerable groups; investing in surveillance technologies and early‑warning systems	Local authorities, community groups, Indigenous organizations
**Long‑term Governance and Global Cooperation**	Adopt due‑diligence laws; integrate toxic‑metal monitoring into climate finance; establish reference guidelines; pursue multilateral agreements	International bodies, regional blocs, national governments

## Discussion

The evidence synthesized in this manuscript illustrates a paradox at the heart of the green transition: while the global push for renewable energy and electrification is essential to curb climate change, it also risks amplifying the toxic metal burdens borne by workers, communities, and ecosystems. This tension is not theoretical. As presenters at the *Toxic Metals Symposium 2025* demonstrated through diverse regional studies, increased mining, metal processing, informal recycling, and weak governance have already begun to create new generations of exposure hotspots alongside long‑standing legacy contamination.

A recurring theme in the discussions was that environmental health, occupational health, and climate mitigation must not operate in silos. As Karin Broberg (Lund University) and Philip Landrigan (Boston College) emphasized, past industrial revolutions left a legacy of leaded petrol, mercury pollution, and unsafe working conditions. Without proactive safeguards, good governance, and adequate funding, the rush for critical minerals may repeat those mistakes, deepening health inequities rather than delivering just, sustainable development.

Findings presented at the symposium confirm that modern supply chains remain leaky. For example, data on cobalt and nickel in battery recycling workers show that even state‑of‑the‑art factories can fail to control exposures if occupational health standards lag behind industrial growth. Informal recycling hubs like Agbogbloshie, Ghana, highlighted by Andrea Kaifie‑Pechmann (FAU Erlangen) and Johanna Elbel (Sciences Po Paris), underscore how marginalized populations face multiple burdens: environmental toxins, lack of healthcare, and economic precarity.

This evidence supports a broader insight: technological solutions alone are insufficient. Advances in biomonitoring, such as Stefan Rakete’s (LMU Munich) micro‑sampling and Don Smith’s (University of California) utilizing multi‑media biomarkers and advanced mass spectrometry methods, are crucial but must be paired with regulatory action and community capacity to interpret and act on data. Likewise, sophisticated supply chain audits and corporate certifications have little impact if informal or artisanal workers remain excluded from legal protections and economic alternatives.

The symposium also clarified key research gaps. While the toxicological effects of lead and mercury are well documented, evidence on emerging metals (e.g., cobalt, nickel nanoparticles, lithium) is still evolving. Long‑term epidemiological studies are needed to assess cumulative, low‑dose, and combined exposures, particularly in vulnerable groups, such as pregnant women and children. Several speakers called for more interventional research to identify what community and policy interventions most effectively reduce exposure in real‑world conditions.

Another challenge is financial sustainability. Many LMICs rely on donor‑funded pilot clean‑ups or monitoring campaigns that often fade once external funding ends. Pamela Mwanza’s (Zambia Environmental Management Agency) experiences in Zambia illustrate how political turnover and budget cuts can derail even well‑planned remediation. Sustainable financing must therefore be integrally embedded in climate finance and green investment frameworks from the beginning, ensuring that the true social and environmental costs of mineral extraction are accounted for upfront.

Finally, the symposium highlighted the critical importance of environmental justice. The communities producing critical minerals are often far removed from those consuming clean energy technologies. As Rachel Bonnifield (Center for Global Development) noted, this geographic and economic disconnect allows powerful actors to externalize environmental health costs onto marginalized populations. The global climate discourse must therefore expand to include commitments to toxic metal mitigation, corporate due diligence, and community empowerment as integral parts of the energy transition.

In sum, the discussion makes clear that addressing toxic metals in the context of decarbonization is not merely a technical problem but a governance and equity challenge. The roadmap presented here offers a starting point, but its success depends on genuine cross‑sectoral collaboration, sustained funding, and political will.

## Conclusions

This article synthesizes the main insights and consensus from the Toxic Metals Symposium 2025, drawing attention to an urgent but often overlooked dimension of sustainable development. While decarbonizing the global economy is non‑negotiable to mitigate climate risks, it must not come at the cost of expanding toxic metal exposure, especially in regions least equipped to manage its consequences.

The evidence is clear: modern and legacy sources of lead, mercury, arsenic, cadmium, uranium, and other metals continue to affect the health of millions of people worldwide. New industrial activities to produce critical minerals risk adding fresh exposure pathways unless robust safeguards are enforced. Health effects—from neurodevelopmental deficits and carcinogenesis to systemic organ damage—are well documented, and new research points to complex, cumulative risks that extend across the life course and potentially across generations.

Encouragingly, technological advances in monitoring, coupled with community‑driven initiatives, offer practical tools to identify and manage risks. Policy gaps, however, remain significant: weak regulatory enforcement, inconsistent standards, informal sectors operating outside legal protections, and limited resources for remediation all undermine progress.

This article proposes a multi‑tiered roadmap to guide policymakers, industry leaders, researchers, and communities. Immediate actions should focus on expanding surveillance, raising public awareness, and addressing acute hotspots. Medium‑term strategies include harmonizing standards, adopting cleaner technologies, and formalizing informal sectors. Long‑term success hinges on binding corporate due diligence, sustainable financing mechanisms, and embedding toxic metal governance into global climate policy frameworks.

Ultimately, a just and sustainable energy transition must ensure that the benefits of climate mitigation do not come at the expense of health and equity of workers and communities. Integrating toxic metal safeguards into climate action plans is both a moral imperative and a practical necessity for achieving the SDGs. The scientific, technological, and policy tools exist; what remains is the collective will to apply them decisively and equitably.

## Figures and Tables

**Table:** 

ADME	(Absorption, distribution, metabolism, and excretion)
As	Arsenic
ASGM	Artisanal and small‑scale gold mining
ASM	Artisanal and small‑scale mining
ASQ‑3	Ages and stages questionnaire
BLL	Blood lead level
Cd	Cadmium
CDC	Centers for Disease Control and Prevention
Co	Cobalt
Cr VI	Hexavalent chromium
Cr	Chromium
CR	Corporate responsibility
CSDDD	Corporate Sustainability Due Diligence Directive
CVD	Cardiovascular disease
DBS	Dried blood spot
EHIA	Environmental and health impact assessment
EV	Electric vehicle
Ge	Germanium
GGT	Gamma‑glutamyl transferase
Hb	Hemoglobin
Hg	Mercury
HIC	High‑income country
IIEF	International Index of Erectile Function
In	Indium
IQ	Intelligence quotient
LDH	Lactate dehydrogenase
Li	Lithium
LiB	Lithium‑ion battery
LMIC	Low‑ and middle‑income country
NGO	Non‑governmental organization
Ni	Nickel
Pb	Lead
PPE	Personal protective equipment
qDBS	Quantitative dried blood spot
REE	Rare earth elements
SDG	Sustainable development goal
U	Uranium
ULAB	Used lead‑acid battery
UN	United Nations
VAMS	Volumetric absorptive microsampling
WHO	World Health Organization
XRF	X‑ray fluorescence
mg/kg	Milligram per kilogram
µg/L	Microgram per liter
µg/dL	Microgram per deciliter
µg/g	creatinine microgram per gram creatinine
µL	Microliter
mmol/L	Millimole per liter
ng/dL	Nanogram per deciliter

## Data Availability

This review did not generate new datasets. All information and data discussed are available in the cited literature.

## Notes

https://www.iea.org/reports/the-role-of-critical-minerals-in-clean-energy-transitions/executive-summary.

https://www.icj-cij.org/sites/default/files/case-related/187/187-20250723-pre-01-00-en.pdf.

https://www.theguardian.com/environment/2026/jan/10/china-mine-europe-rare-earth-metals-swedish-producer.

https://www.nytimes.com/interactive/2025/11/18/world/africa/lead-poisoning-car-battery.html?smid=nytcore-ios-share&referringSource=articleShare.

https://www.icj-cij.org/sites/default/files/case-related/187/187-20250723-pre-01-00-en.pdf.

## Verification

All authors had access to the data. They also had a role in writing the manuscript.

## Additional File

The additional file for this article can be found as follows:
