# Supplementary material for: Toxic Metals in a Green Transition: Global Health Risks, Sources, and Policy Responses—Insights from the Munich Toxic Metals Symposium 2025
Source: Ann Glob Health. 2026 Apr 7;92(1):29. doi: 10.5334/aogh.5214 (PMC13068094; doi:10.5334/aogh.5214)
Supplement: Supplementary File 1. — Supplementary files. [file agh-92-1-5214-s1.pdf]

Supplement

Abstracts (alphabetical order family name)

AB Alilović: Mercury exposure via tuna consumption

Adna Alilović - Institute Jožef Stefan Slovenia

Adna Alilović is a PhD student at the Department of Environmental Sciences, Jožef Stefan Institute in Ljubljana, Slovenia. Her research focuses on human health risk assessment related to mercury exposure.

All authors and affiliations

Adna Alilović (1,2); Janja Snoj Tratnik (1,2); Polona Klemenčič (2); Marta Jagodic Hudobivnik (2); Ingrid Falnoga (2); Darja Mazej (2); Igor Živković (1,2); Milena Horvat (1,2)

1. International Postgraduate School Jožef Stefan, Ljubljana, Slovenia
2. Jožef Stefan Institute, Department of Environmental Sciences, Ljubljana, Slovenia

Background

Fish is a valuable source of omega-3 fatty acids, vitamins, and minerals. However, it is also the primary source of mercury (Hg) exposure in the general population, particularly methylmercury (MeHg), which accumulates in large predatory species like tuna. Health risk assessments often assume that all Hg in fish is MeHg and that 95–100% of ingested MeHg is bioavailable. Recent studies challenge these assumptions, suggesting they may overestimate exposure and misrepresent the risk–benefit balance of fish consumption. Fish also contain selenium (Se), which may mitigate MeHg toxicity, though its influence on MeHg kinetics under realistic dietary conditions in humans remains unclear.

Methods/Approach

The present study investigated Hg and Se biomarker changes in humans following controlled tuna consumption. Ten individuals consumed five tuna steaks over five days, six controls abstained from seafood. Total Hg (THg), MeHg, and Se were measured in all steaks. Regular blood and urine sampling enabled tracking of THg, MeHg, and Se kinetics during exposure and elimination.

Results

MeHg comprised an average of 84% (range: 69–98%) of total Hg in the tuna. Se concentrations in steaks were positively correlated with Hg. In exposed individuals, THg and MeHg levels in whole blood, plasma, and erythrocytes increased linearly with dose, peaking after the fifth steak and subsequently declining in a biphasic manner. Urinary THg showed a delayed increase, reaching maximum about one

month post-exposure, consistent with ongoing MeHg demethylation. Plasma and urinary Se increased during the exposure, quickly returning to baseline within 10 days post-exposure, while erythrocyte Se remained elevated throughout the study. Correlations between Hg and Se biomarkers in plasma and urine shifted over time, from positive during exposure to negative during post-exposure.

Conclusions and next steps

Despite continuing research, Se-Hg interactions in humans are still not fully understood, and further efforts are needed to unravel the intricate dynamics at play.

Email contact

milena.horvat@ijs.si

Funding source

ARIS programme P1-0143; ARIS projects: J7-9400 NEURODYS, J7-60123 MultiPart; National HBM Program GA C2715-20-634801; AlpaCem d.o.o. Anhovo fellowship

AB Bose-O'Reilly: Artisanal and Small-Scale Gold Mining in Sub Saharan Africa and Children's Health

Stephan Bose-O'Reilly - Institute and Clinic for Occupational-, Social-, and Environmental Medicine, University Hospital, LMU Munich Germany

Stephan Bose-O´Reilly, is a professor for Environmental and Public Health at the University Hospital, LMU Munich. He is a paediatrician whose main interest is to prevent children from disease by helping to reduce their exposure to toxic substances, such as lead and mercury. His special interests are introducing micro-sampling methods, transferring knowledge and capacities, and training of scientists and experts to improve children's environmental health.

All authors and affiliations

Stephan Bose-O'Reilly (1)

1. Institute and Clinic for Occupational-, Social-, and Environmental Medicine, University Hospital, LMU Munich, Ziemssenstr. 5, D-80336 Munich, Germany

Background

Artisanal and small-scale gold mining (ASGM) supports the livelihoods of millions across Sub-Saharan Africa (SSA), including in Ghana, Tanzania, Kenya, Senegal and Zimbabwe. However, the sector is the largest global source of mercury pollution, contributing over 38% of global emissions. Mercury exposure presents acute and chronic health risks, particularly for children, who are highly susceptible due to their developing nervous systems. This study examines the health impacts of mercury exposure on children in ASGM communities across the SSA countries.

Methods/Approach

A review of peer-reviewed literature, national reports, and environmental health assessments was

conducted to synthesize data on mercury use in ASGM and its implications for child health. Studies were selected based on relevance to child and maternal health outcomes in proximity to ASGM activities. Biomonitoring data and health statistics were evaluated comparatively.

Results

Children in ASGM areas are exposed to mercury both directly—through inhalation of vapor during amalgamation—and indirectly, via contaminated food and water. Children living near ASGM sites exhibited elevated mercury levels in blood, hair and urine, with associated cognitive and developmental impairments. Children working as amalgam burners showed clinical mercury intoxication, with children in surrounding communities experiencing significant neurodevelopmental delays. Across all countries, mercury exposure has been linked to increased incidence of stillbirths and adverse birth outcomes among women. Limited access to healthcare, protective equipment, and sanitation compounds these risks.

Conclusions and next steps

Mercury use in ASGM poses a serious threat to child health in Sub-Saharan Africa. Immediate policy attention is needed to eliminate mercury use, strengthen health monitoring, and ensure targeted interventions. Protecting children in mining communities requires coordinated efforts across public health, mining regulation, and community engagement sectors.

Email contact

stephan.boeseoreilly@med.uni-muenchen.de

Funding source

World Bank

AB Bonnifield: "Do No Harm": Understanding and Addressing Lead Exposure in Development Programs

Rachel Bonnifield - Center for Global Development The United States of America (USA)

Rachel Bonnifield is Director of Global Health Policy and Senior Fellow at the Center for Global Development, a non-profit, non-partisan, independent think tank based in Washington, D.C. and London, working on issues that affect the poor in low- and middle-income countries and our shared global future.

All authors and affiliations

Rachel Bonnifield (1)

1. Center for Global Development

Background or Purpose

Roughly half of children in low- and middle-income countries (LMICs) have blood lead levels (BLLs) exceeding the WHO's threshold for clinical intervention, with enormous implications for their health, cognitive development, and long-term life outcomes. Counterintuitively, many development programs

that seek to address these same challenges may also inadvertently introduce lead hazards, for example lead acid batteries for energy storage or lead paint in infrastructure.

Content

This presentations considers the intersection between finance and interventions -- in education, energy, climate, and so forth -- and risks and mitigation opportunities related to childhood lead exposure.

Implications for addressing the issue

Development institutions and funders should ensure a "do no harm" approach to all cross-sectoral programs to protect against inadvertent resultant lead exposure in intended beneficiaries and communities.

Potential follow-up/actions

Development institutions and funders should ensure a "do no harm" approach to all cross-sectoral programs to protect against inadvertent resultant lead exposure in intended beneficiaries and communities.

Email contact

rbonnifield@cgdev.org

Funding source

Open Philanthropy

AB Elbel: Dynamics of value and power: Organization of the informal e-waste sector in Ghana and its health impacts

Elbel Johanna - Sciences Po Paris France

Johanna Elbel holds a Master's in Public Policy and Global Health and has previously gained professional experience in the politics of global health policy. Her research centres on environmental and occupational health, with a specific interest in the political and commercial determinants of health of mineral extraction and global supply chains.

All authors and affiliations

Johanna Elbel (1)

1. School of Public Affairs, Sciences Po University, Paris; Adress: Sciences Po, 1 place Saint-Thomas d’Aquin, 75007, Paris.

Background or Purpose

Ghana’s informal e-waste sector sits at the intersection of global waste and recycling value chains and is central to circular economy narratives. The sector faces severe environmental and health risks, and policy approaches to regulate these risks differ. This study investigates decision-

making processes in Ghana’s e-waste sector, focusing specifically on the power dynamics that shape policy and its effects on workers’ health.

Content

The case study showcases the potential for the sustainable transformation of the Ghanaian e-waste sector within a multi-stakeholder landscape. These stakeholders are setting up a novel approach to the organization of the e-waste sector and its environmental hazards, which is based on incentive-based mechanisms. In addition, e-waste workers, are returning to the scrapyard Agbogbloshie in 2025 after its destruction in 2021, highlighting resistance to coercive, top-down approaches. Differentiating policy strategies can be identified. The success of policy depends on the recognition of informal e-waste workers, which influences environmental policy compliance and health outcomes.

Implications for addressing the issue

Informal e-waste workers’ health can be positively impacted by a strengthened multi-stakeholder e-waste management system that addresses workers’ needs. By strengthening workers’ associations and increasing the willingness of workers to join these, the participation and reach of collectors can be ensured. To organize the system in Ghana sustainably, donor dependency must be decreased, potentially by expanding national recycling capacities. General awareness of e-waste as a problem, along with its environmental and health effects, must be improved.

Potential follow-up/actions

Future research on the willingness of workers to shift to the organized sector and on the willingness of the public to participate in collection systems is recommended. Further, a comparison of private versus public initiatives and their different potential to impact occupational and environmental health should be explored to develop best-practice examples.

Email contact

johanna.elbel@sciencespo.fr

AB Fatriah**: Criminal Arsenic Poisoning, a Case Study**

Dr. Syarifah Hidayah Fatriah, SpFM - Faculty of Medicine,

Universitas Muhammadiyah Riau Indonesia

Dr. Syarifah Hidayah Fatriah, SpFM was born in Pekanbaru on May 18, 1988. She is a graduate of the Forensic and Medicolegal Medicine Program at the Faculty of Medicine, University of Indonesia. Currently, she is a lecturer and the Vice Dean for Academic and Student Affairs at the Faculty of

Medicine, Universitas Muhammadiyah Riau, and also forensic

pathologist at Santa Maria Hospital in Pekanbaru.

All authors and affiliations

Syarifah Hidayah Fatriah (1), Muchtaruddin Mansyur (2)

1. Department of Forensic Medicine of Medicine, Universitas Muhammadiyah Riau, Indonesia

2. Dept. of Community Medicine, Faculty of Medicine, Universitas Indonesia, Jakarta Indonesia

Background

Arsenic poisoning is a severe form of heavy metal toxicity that can be fatal if not diagnosed and treated in time.As a naturally occurring element, arsenic is widely utilized in industrial applications and pesticides, making human exposure possible through ingestion, inhalation, or skin contact. The toxic effects of arsenic stem from its ability to disrupt essential cellular processes, leading to mitochondrial dysfunction, oxidative stress, and cell death. Forensic investigations play a crucial role in identifying arsenic poisoning. This paper explores the mechanisms of arsenic toxicity, clinical symptoms, and forensic approaches in detecting arsenic-related deaths, emphasizing the importance of forensic toxicology in legal investigations.

Methods/Approach

This study reviews existing literature on arsenic poisoning, including its toxicokinetics, clinical manifestations, and forensic detection methods. Information is gathered from toxicological reports, autopsy findings, and exposure investigations to understand the impact of arsenic poisoning on the human body and its role in forensic medicine. Case studies provide real-world insights into the forensic analysis required to confirm arsenic-related fatalities.

Results

Acute arsenic poisoning presents with severe gastrointestinal distress, cardiovascular complications, and neurological dysfunction, progressing rapidly to multiorgan failure and death. Forensic investigations utilize toxicological analyses, autopsy results, and exposure histories to confirm arsenic poisoning as the cause of death. The use of arsenic as a tool for homicide highlights the need for thorough forensic examination to establish cause-of-death findings.

Conclusions and next steps

Arsenic poisoning remains a significant challenge due to its widespread industrial use and potential for intentional misuse. Understanding its toxic effects and forensic detection methods is essential for medical professionals, legal authorities, and public health experts. Proper investigation and toxicological analysis are crucial in identifying arsenic poisoning cases, preventing exposure, and ensuring justice in forensic and legal proceedings. The forensic assessment of arsenic-related deaths reinforces the need for multidisciplinary collaboration in forensic toxicology to uphold truth and legal integrity.

Email contact

syarifahhidayahfatriah@umri.ac.id

AB Fitriani: Occupational and Environmental Exposure to Lead in Indonesia

Dewi Yunia Fitriani - Universitas Indonesia Indonesia

Dr. Dewi Yunia Fitriani is an assistant professor in the Department of Community Medicine, Faculty of Medicine, Universitas Indonesia, and an occupational medicine physician at Universitas Indonesia Hospital. She currently leads the Occupational and Environmental Health Research Center (OEHRC) at the Indonesian Medical Education and Research Institute (IMERI). Her academic interests include mental health among workers, toxic metal exposure, and occupational health surveillance.

All authors and affiliations

Muchtaruddin Mansyur (1,2); Dewi Yunia Fitriani (1,2); Ari Prayogo (1,3); Ade Mutiara (1,2); Asep (1,2); Ratih Fadhillah (1,2); Rifka Aini (1,2); Winda Widyaning Putri (1,2); Sarah Edna Fadilah Ramadhani (1,2); Agus Kharmayana Rubaya (5); Sarjito Eko Windarso (4); Herman Santjoko (4); Sigid Sudaryanto (4); Haryono (4); Budi Susilorini (5); Nickolaus Hariojati (5); Alfonso Rodriguez (6); Stephan Bose O'Reilly (7)

1. Occupational & Environmental Health Research Centre, Indonesian Medical and Education Research Institute (IMERI), Faculty of Medicine, Universitas Indonesia, Central Jakarta, Indonesia
2. Department of Community Medicine, Faculty of Medicine, Universitas Indonesia, Central Jakarta, Indonesia
3. Tebet Regional Public Hospital, South Jakarta, Indonesia
4. Health Polytechnic of Ministry of Health, Yogyakarta, Indonesia
5. Yayasan Pure Earth Indonesia, Victorian Business Park, Banten, Indonesia
6. Pure Earth, 475 Riverside Drive, New York, USA
7. Institute and Clinic for Occupational, Social and Environmental Medicine, University Hospital, LMU Munich, Germany

Background

Indonesia continues to face a significant public health threat from lead (Pb) exposure, primarily due to informal recycling of used lead-acid batteries (ULABs), unregulated metalworking industries, and widespread environmental contamination. This presentation shares findings from recent field studies that examined both occupational and environmental pathways of lead exposure in four high-risk communities across Java Island.

Methods/Approach

This cross-sectional study assessed BLL levels in children aged four in communities exposed to used lead-acid battery (ULAB) recycling activities, comparing them to a control area. The study employed a threshold level of 20 µg/dL to identify high BLLs and utilized a sample size of 324 children from exposed sites and 240 from control. Data collection was carried out through questionnaires, laboratory methods, home-based assessment (HBA), and sampling households. Determining BLL, the capillary blood sample was analysed using LeadCare II which detected BLL ranging from 3.5 to 65.0

µg/dL. This study established a cut-off point for blood lead levels at 20 µg/dL, as haem synthesis is known to be disrupted at this threshold.

Results

Results showed that 29% of the children had BLLs ≥5 µg/dL, with several cases exceeding the LeadCare II analyser's maximum detection limit of 65 µg/dL. Environmental sampling revealed median soil lead concentrations of 6,582 ppm in exposed areas, well above national and WHO safety thresholds. Determinants of elevated BLLs included parental occupational exposure, use of aluminium cookware and contaminated spices, as well as poor household hygiene practices. A strong association was also observed between paternal and child BLLs, which reinforces the importance of take-home exposure pathways.

Conclusions and next steps

Despite the clear health risks, Indonesia currently lacks a national surveillance or remediation program to address lead exposure. These findings emphasize the need to implement integrated environmental and occupational health policies, targeted risk communication, and scalable biomonitoring and remediation strategies.

Email contact

dewi.yunia.fitriani@gmail.com

AB Flores: Artisanal gold mining, Mercury and Health. Paraguay

Laura Emilce Flores Rodriguez - Hospital de Clínicas Paraguay

Dr. Flores is chair of the Department of Occupational Health at Clinic Hospital. Medical doctor since 1997, specialized in Internal Medicine since 2002. Specialized in Occupational Medicine since 2007(Buenos Aires, Argentina). Master in Preventions of Occupational Hazards, Industrial Hygiene since 2010 (OISS/University of Alcala de Henares. Spain). Master in Sciences in International Occupational Safety and Health since 2016 (Munchen University. Germany). Fellowship in Environmental Health (Corea del Sur).

All authors and affiliations

Laura Flores (1); Patricia Giménez (2); Patricia Alfonso (3); Gloria León (4); Ovidio Espinola (5); Mauricio Rodas (6);, Maria Irene Santacruz (7); Margarita Correa (8); Rocio Ramirez (9)

1. Hospital de Clínicas, San Lorenzo, Paraguay
2. MADES, San Lorenzo, Paraguay

Background

Artisanal gold mining is considered one of the extractive processes with greater amount of environmental problems, and to human health, it is calculated that for every gram of gold mined, 7 to 30 grams of mercury can be used. The inappropriate use of mercury has neurotoxic effects and

systemic in both occupational and environmental exposures. Objectives: Evaluate the health impacts of mercury use in artisanal miners in Yobai pass in the years 2018-2019.

Methods/Approach

Cross-sectional, observational, descriptive study. 99 miners of Paso Yobau were evaluated. A) Occupational clinical history was applied b) Questionnaire of Neurotoxic Symptoms Q 16 c) Mini Mental test d) study tremographic e) Biological Surveillance Mercury in 24-hour urine. The data were analysed with SPSS 25.

Results

78.2% were male, mean age 34.1 ± 12.9. 62.2% had studies basic. 61.4% handle mercury. The mean years 7.4 ± 5.8 years. 20.2% they had arterial hypertension, abortion 10.1% and congenital malformation in 6.7%. Use of mask in 46.1%, gloves in 40.2%, boots in 17.1%. Trembling fingers 23.2%, nausea and vomiting in 22.8%. Questionnaire Q16 with disorders of memory 42.4%, change of mood and anger 39.4%, paresthesias 29.2%. Chi: 0.012 in the group that handles mercury. Mini mental Test 39.8% with slight impairment, 15.3% are with moderate deterioration. Mouth ulcers in 27.3%, gingivitis in 13.1%, halitosis in 10.1%, gingival border in 8.1%. Skin lesions 6%, tremor by 33%. The mean Hg concentration 2.21 ± 2.30 ug / 24 hours, 1.1% exceed the value. Those who handle mercury present values equal to and / or above 5 ug / 24 hours, p = 0.006 (Chi Square).

Conclusions and next steps

Multiple social, economic, educational, and labor factors are determining factors of the state of health, for which the integration of the different actors for an adequate system of prevention and health promotion in the Yobai Pass community.

Email contact

floreslaurapy@yahoo.com

Funding source

Ministry of the Environment

AB Freixas: Diet dependence of mercury exposure in Northern and Southern European populations

Martí Nogués Freixas - IDAEA - CSIC Spain

Martí Nogues Freixas is a PhD student in Analytical and Environmental Chemistry at the Institute of Environmental Assessment and Water Research (IDAEA-CSIC) in Barcelona, Spain. His research focuses on the analysis of metals, particularly mercury, in various types of samples, including both environmental matrices (fish, sediments, and water) and human biological samples (hair and urine).

All authors and affiliations

Martí Nogués (1,2); Joan O. Grimalt (1); Mercè Garí (1); Adonina Tardón (3); Kinga Polanska (4)

1. Institute of Environmental Assessment and Water Research (IDAEA – CSIC), Barcelona, Spain

2. Faculty of Chemistry, Universitat de Barcelona (UB), Barcelona, Catalonia, Spain
3. Unidad de Molecular Epidemiology, University Institute of Oncology of the Asturias Principality (UIOPAÇ), Oviedo, Spain
4. Department of Environmental and Occupational Health Hazards, Nofer Institute of Occupational Medicine (NIOM), Lodz, Poland

Background
Mercury exposure represents a significant public health issue, particularly through the consumption of contaminated fish. Mercury in hair is used as a biomarker of internal exposure. This study analyse s mercury concentrations in hair samples from two populations: adults from the Metropolitan Area of Barcelona (Catalonia, Spain, n=45) and teenagers from the REPRO_PL birth cohort (Poland, n=80), and compares these data with additional measurements from Menorca (Balearic Islands) and Flix (Catalonia). Significant correlations between hair concentrations and diet using food frequency questionnaires are investigated to identify the exposure sources of mercury in the studied cohorts.

Methods/Approach
Hair samples were collected 1–2 cm from the scalp and pre-treated with successive washes of hexane, Triton X-100, and Milli-Q water to remove external contamination. Total mercury (THg) was measured using DMA-80, which allows precise determination in solid samples without chemical treatments. Food frequency questionnaires were administered to the families of the participant families.

Results
The median concentrations of THg in the Polish teenagers was 72 µg/kg, ranging from 3.9 µg/kg to 1500 µg/kg. In contrast, the median concentration in adults from Barcelona was 989 µg/kg, ranging from 138 µg/kg to 4424 µg/kg.

Conclusions and next steps
The differences in THg concentrations found between Barcelona and Poland are probably related to fish consumption, since Mediterranean populations have higher intakes than central Europe, in line with previous findings (Smolders et al., 2015). Further analysis is required to confirm these findings, including the determination of larger number of samples and the evaluation of fish consumption.

Smolders, R., et al. (2015). Interpreting biomarker data from the COPHES/DEMOCOPHES twin projects: Using external exposure data to understand biomarker differences among countries. Environmental Research, 141, 86–95.

Email contact
marti.nogues@idaea.csic.es

AB Harari: Exposure to metals during recycling of lithium-ion batteries for electric vehicles

Florencia Harari Thuresson - School of Public Health and Community Medicine, University of Gothenburg Sweden Dr. Harari is an Associate Professor at the School of Public Health and Community Medicine, University of Gothenburg, and Resident Physician in Occupational and Environmental Medicine at the Sahlgrenska University Hospital. Dr. Harari obtained her MD in Ecuador in 2010 and

her PhD degree at Karolinska Institutet. Dr. Harari conducts research on occupational and environmental exposures and health effects in human populations, with special focus on metals and chemical substances.

All authors and affiliations Florencia Harari (1); Erik Rosengren (1); Klara Midander (2); Anneli Julander (2); Sandra Johannesson (1) 1. Occupational and Environmental Medicine, School of Public Health and Community Medicine, University of Gothenburg, and Sahlgrenska University Hospital, Gothenburg, Sweden. 2. Swedish Environmental Research Institute (IVL), Sweden.

Background Lithium-ion batteries (LiB) are essential for electrification needed to achieve the climate goals, thus large-scale LiB manufacturing and recycling facilities are emerging globally. This pilot study aimed at characterizing metal exposure through air, skin and biomonitoring at a new recycling facility of LiB from electric vehicles.

Methods/Approach Biomonitoring (blood, plasma and urine) was performed in 16 employees at two time points: baseline (before start of production) and follow-up (four months later). Among these, five workers (from separation and sorting n=3, storage n=1, and maintenance n=1) were selected to perform exposure assessment through air using personal air samplers for inhalable dust, through skin by acid-wipe sampling, and biomonitoring (pre- and post-shift and pre-shift one day later). All samples were analysed for lithium, nickel and cobalt, by inductively coupled plasma mass spectrometry.

Results Airborne metal concentrations exceeded occupational exposure limits for all three workers in separation and sorting (work-shift averages, in µg/m3: lithium 17-78, nickel 85-351, cobalt 25-117). Direct reading particle instruments showed high peak exposures of dust during specific work tasks. Inhalable metal concentrations were low among storage and maintenance workers. Nickel (0.002-0.3 µg /cm2) and cobalt (0.001-0.1 µg /cm2) were detected on the hands of the five workers. Among the 16 workers, metal concentrations in blood, plasma and urine were low, similar to those found in the general population.

Conclusions and next steps Low metal concentrations in biological samples among workers in sorting and separation, where metal dust concentrations in air were elevated, suggest effective use of powered air purifying respirators. Sensitizing metals were detected on skin of all workers, in some cases at ranges that trigger eczema in sensitized individuals. Efforts to reduce metal emissions at the sources are needed.

Email contact Florencia.harari@amm.gu.se

Funding source Swedish Research Council for Health, Working Life and Welfare (Forte) and Afa Försäkring.

AB Hartwig: Toxicology of Aluminium

Andrea Hartwig - Karlsruhe Institute of Technology (KIT), Food Chemistry and Toxicology Germany

Dr. Andrea Hartwig is professor for Food Chemistry and Toxicology at the KIT, specialized on molecular mechanisms of metal toxicology. Furthermore, she is chair of the German MAK commission and thus involved in toxicological risk assessment.

All authors and affiliations

Andrea Hartwig (1)
1. Karlsruhe Institute of Technology (KIT), Institute of Applied Biosciences (IAB), Food Chemistry and Toxicology, Karlsruhe, Germany

Background

Aluminium is a naturally occurring element. Major sources of human exposure are food additives, manifold consumer products, such as Al containing antiperspirants, and vaccines. Furthermore, workplace exposure via inhalation of aluminium compounds is of high relevance.

Methods/Approach

The MAK commission has reviewed all available literature, thereby identifying adverse health effects after different routes of exposure and considering different aluminium compounds.

Results

While absorption via the intact skin and the gastrointestinal tract is rather low, inhalative exposure occurring at workplaces, but also in case of respective antiperspirant sprays is of high relevance. Critical toxicological endpoints are neurotoxicity, irritation as well as inflammatory responses, depending on solubility and particle size, resulting in differences in local and systemic bioavailability.

Conclusions and next steps

To protect from adverse health effects, different threshold values for Al in air as well as in biological media have been established for different Al compounds. Even though frequently discussed, there is no consistent evidence on the development of breast cancer through the use of aluminium-containing antiperspirants.

Email contact

Andrea.Hartwig@kit.edu

AB Issah: Metal exposures and biomarkers of liver damage: A systematic review of observational studies

Ibrahim Issah - University of Ghana

Dr. Ibrahim Issah's research and teaching interests focus on occupational health and safety, environmental exposure assessment, toxic metals, and environmental epigenetics. His work bridges theoretical frameworks and applied field studies, particularly in understanding how occupational and environmental exposures affect human health.

All authors and affiliations

Ibrahim Issah (1,2); John Arko-Mensah (1,2); Mabel S. Duah(1,2); Serwaa A. Bawua (1,2); Shirley V. Simpson (2,3); Thomas P. Agyekum (4); Olalekan Uthman (5), Julius N. Fobil (1,2)

1. Department of Biological, Environmental and Occupational Health Sciences, University of Ghana,

School of Public Health, Accra, Ghana;
2. West Africa Center for Global Environmental & Occupational Health, College of Health Sciences, University of Ghana, Legon, Accra, Ghana
3. Department of Bacteriology, Noguchi Memorial Institute for Medical Research, University of Ghana, Legon, Ghana;
4. Department of Occupational and Environmental Health and Safety, School of Public Health, College of Health Sciences, Kwame Nkrumah University of Science and Technology, Kumasi 00233, Ghana
5. Warwick Applied Health, Warwick Centre for Global Health, Warwick Medical School, University of Warwick, Coventry, CV4 7AL, United Kingdom

Background

Exposure to toxic metals is a significant environmental and occupational health concern, with potential implications for liver function. Although individual studies have examined the association between metals exposure and liver damage biomarkers, a comprehensive synthesis of evidence is needed to estimate the overall prevalence, observe trends, and understand the relationship with metal exposures. This systematic review evaluated the association between exposure to metals and key biomarkers of liver damage.

Methods/Approach

This systematic review was conducted following the PRISMA 2020 guidelines and was registered in PROSPERO (CRD42024608273). A comprehensive literature search was performed in PubMed, Scopus, and CINAHL Complete. Studies were included if they were peer-reviewed observational research articles published in English, assessed environmental or occupational exposure to metals, as measured in biological tissues or fluids, and reported any liver damage biomarker (ALT, AST, ALP, and GGT). Quality assessment was conducted using the Newcastle-Ottawa Scale. A narrative synthesis was performed to summarize the findings.

Results

A total of 39 studies met the inclusion criteria. The studies primarily originated from North America, Europe, and Asia, with no studies from Africa. The toxic metals: cadmium (Cd), lead (Pb), mercury (Hg), and arsenic (As) were significantly associated with elevated liver enzyme levels. Most studies identified positive associations between metal exposure and biomarkers of liver injury, with variations based on the exposure levels. Essential metals such as zinc (Zn) and selenium (Se) showed protective effects at physiological levels but contributed to liver dysfunction at high body concentrations. In addition, studies that employed mixture analysis methods, such as Bayesian Kernel Machine Regression (BKMR) and Weighted Quantile Sum (WQS), seem to suggest that the combined effects of multiple metals may exacerbate hepatotoxicity.

Conclusions and next steps

This review strengthens the evidence linking metals exposure to liver dysfunction or damage and underscores the need for targeted regulatory measures to mitigate exposure risks.

Email contact

iissah@ug.edu.gh

AB John: Lead exposure of children in a former mining area in Germany

Lea John - Institute and Clinic for Occupational, Social and Environmental Medicine Germany

Lea John is a PhD candidate at the Institute and Clinic for Occupational, Social and Environmental Medicine

All authors and affiliations

Lea John (1); Dr. Laura Wengenroth (1); Dr. Stefan Rakete (1); Prof. Dr. Dennis Nowak (1); Prof. Dr. Katja Radon (1)

1. Institute and Clinic for Occupational, Social and Environmental Medicine

Background

The district of Goslar, Germany, is historically shaped by intensive mining activities, resulting in long-term environmental contamination—particularly with lead. While typical background concentrations of lead in soils in Lower Saxony, Germany range between 20–40 mg/kg, parts of Goslar, Lower Saxony, Germany exhibit median values exceeding 1,500 mg/kg. Children are especially vulnerable to lead exposure, mainly through hand-to-mouth contact with contaminated soil and dust during outdoor activities. Even low blood lead levels (BLLs) can adversely affect neurodevelopment.

Methods/Approach

The BLENCA2 study aimed to assess current BLLs among preschool children in Goslar and to identify exposure pathways and regional disparities. Of approximately 1,200 children undergoing school-entry health exams, 310 (26%) participated in the study (Sep 2023 – Jun 2024). Blood samples were analysed for lead; caregivers completed questionnaires and interviews.

Results

The geometric mean BLL was 23 µg/l, with 51% of children exceeding age-specific German reference values (22 µg/l for boys, 19 µg/l for girls), 24% exceeding age-specific US reference values (35 µg/l) and 13% above the WHO intervention threshold of 50 µg/l. Higher BLLs correlated with elevated soil contamination at children's residential and recreational locations. Risk factors included lack of regular handwashing and passive smoking.

Conclusions and next steps

These findings emphasize the need for structural, environmental, and behavioural interventions to sustainably reduce lead exposure in children in legacy mining regions.

Email contact

Lea.John@med.uni-muenchen.de

AB Joshi: Heavy metals monitoring following massive use of fire crackers in the city of Delhi

Tushar Joshi - Maulana Azad Medical College India

Tushar Joshi is an adviser to Centre for Occupational and Environmental Health at Prestigious Medical School in Delhi. He set up this first academic centre in India following his return from London School of Hygiene where he was a visiting research fellow. He is a vesting professor at Dornsife School of Public Health, Philadelphia. His centre collaborated with Berkeley School of Public Health and other institutions of excellence to train physicians in the discipline.

All authors and affiliations
Authors: T K Joshi (1); Govind Marwari (2)

Abstract

India celebrates Diwali, a major Hindu festival either in late October or early November when deteriorating air quality due to meteorological conditions favours a rise in air pollution. The toxic emissions due to fire cracker use, compound the air pollution pushing it to hazardous levels. Firecrackers may contain potassium nitrate, sulphur, aluminium and salts of arsenic, aluminium, strontium, manganese, and barium. Rising public concern triggered this study involving a total of 707 and 681 residents from 4 residential areas in pre- and post- Diwali period respectively.
Air quality data was obtained from the national grid of monitoring stations. A questionnaire survey was administered. Urine samples were collected for biological monitoring of metals and were analysed with creatinine correction. The mean levels of all heavy metals were significantly higher in the post-Diwali period as indicated by analysis of urine samples. The mean levels of heavy metals lead, copper, strontium, potassium and magnesium were significantly higher in post-Diwali urine samples. Lead is not present in firecrackers and could be due to other sources.

Background

The city of Delhi in India is rated as one of the most polluted city with hazardous poor quality specially in winter months. This time around a major festival is celebrated where there is massive use of fire crackers. This further aggravates the poor air quality having serious consequence for human health. The rising public concern necessitated this study.

Methods/Approach

This was a field survey that was carried out before and after the celebration of the festival. Urine samples were collected before the onset of the festivities and after the festivities were over. Samples were analysed with ICPMS with creatinine correction.
Air quality data was obtained from the national grid of monitoring stations.

Results

The air quality had deteriorated in post festivities period as compared with levels before the onset of the festivities. There was significant rise in heavy metal levels as detected in urine samples as confirmed by analysis of these samples in post festival period.

Conclusions and next steps

The study demonstrated that massive use of fire crackers add to the already hazardous air quality. The heavy metals which are contained in some of the firecrackers create an exposure scenario where the levels in post festival period register an increase as compared with the pre festival phase.

Email contact

kantjoshi@gmail.com

Funding source

The Pollution Board, A government Entity

AB Kaifie-Pechmann E-waste and occupational health in Ghana

Andrea Kaifie-Pechmann - Institute and Outpatient Unit for Occupational, Social, and Environmental Medicine, Germany

Prof. Kaifie is head of the Institute and Outpatient Unit for Occupational, Social and Environmental Medicine. She focusses in her work on the exposure to hazardous substances in challenging working conditions.

All authors and affiliations

Andrea Kaifie-Pechmann (1)

1. Institute and Outpatient Unit for Occupational, Social, and Environmental Medicine, FAU Erlangen-Nuremberg, Germany

Background

The rapid expansion of electronic technology has led to a parallel surge in electronic waste (e-waste), posing significant environmental and public health challenges globally. In Ghana, particularly in informal recycling hubs such as Agbogbloshie in Accra, the processing of e-waste has become a widespread livelihood, often carried out under unsafe and unregulated conditions.

Methods/Approach

E-waste workers from the Agbogbloshie recycling site as well as a non-exposed control group was recruited and blood as well as urine samples were taken in different study waves. HPLC-ICP-MS as well as AAS was used in order to determine the blood and urine exposure levels.

Results

E-waste workers showed higher median concentrations of lead as well as inorganic arsenic species in comparison to a control group. More than 80% of the e-waste workers exceeded the acceptable concentration of the sum of carcinogenic inorganic arsenic species (14 µg/L), which was significantly higher in comparison to the control group (70%). The tolerable concentration (40 µg/L) was exceeded in 17.2% of the participants.

Conclusions and next steps

This presentation highlights the urgent need for stricter regulatory frameworks, improved waste management infrastructure, and targeted public health interventions to mitigate occupational risks.

Email contact

andrea.kaifie-pechmann@fau.de

AB Keshk: From Artisanal to Industrial-Scale Gold Mining: Marine Mercury Contamination in Two Case Studies from the Global South

Omar Keshk - GEOMAR Helmholtz Centre for Ocean Research Kiel Germany

Omar Keshk is a PhD student at GEOMAR Helmholtz Centre for Ocean Research, studying the impact of mining on the marine biogeochemical cycle of mercury. With a background in environmental chemistry, his academic training spans both scientific and social dimensions of pollution, integrating interdisciplinary approaches to understand and address mercury contamination.

All authors and affiliations

From Artisanal Gold to Industrial-Scale Mining: Marine Mercury Contamination Linked to Mining in the Global South

Omar Keshk (1); Prof. Dr. Christian Winter (2); Prof. Dr. Julia Gottschalk (2); Prof. Dr. Daniel Rosado (3); Dr. Lars-Eric Heimbürger-Boavida (4); Dr. Philipp A. Brandl (1); Dr. Rebecca Zitoun (1) (5); Dr. Gitta Ann von Rönn (2); Dr. Natalia Torres-Rodriguez (4); Jannes Kowalski (2); Prof. Dr. Sylvia Sander (1,2)

1. GEOMAR Helmholtz Centre for Ocean Research, Kiel, Germany
2. Kiel University (CAU), Kiel, Germany
3. University of Seville, Seville, Spain
4. Mediterranean Institute of Oceanography (MIO), Marseille, France
5. University of Tasmania, Institute of Marine and Antarctic Studies, Battery Point, Australia

Background

This study examines mercury (Hg) contamination in sediments from two mining-impacted marine regions. The first site, near the Magdalena River mouth in Colombia might be contaminated with Hg used in artisanal gold mining. The second, in the deep sea near Papua New Guinea (PNG), shows signs of contamination likely linked to mine tailings disposal from the Lihir gold mine. By analysing total mercury (THg) concentrations in sediments and porewaters, the study explores Hg behaviour across coastal and deep-sea environments. This comparison highlights how mining and sediment characteristics influence Hg's fate, enhancing the understanding of Hg contamination in coastal and deep-sea ecosystems.

Methods/Approach

Two box cores were collected during expedition MSM112 with RV Maria S. Merian from the Magdalena Canyon, Colombia, and analyse d for THg. These cores were dated using excess ^{210}Pb profiles to establish a high-resolution sediment chronology. A gravity core from the same area was also analyse d for THg, with radiocarbon dating underway to refine age models and splicing the cores. At the deep-sea site in PNG, one push core and one gravity core, collected during expedition SO299 with RV SONNE, were analyse d for THg and trace metals.

Results

In Colombia, THg concentrations in both box cores have doubled over the past 150 years. The core collected within the river plume exhibited higher THg levels than the one farther from the delta, indicating the plume as the primary contamination source. At the PNG site, both the push core and gravity core showed increased Hg and trace metals in surface sediments, reflecting potential contamination from mine tailings. tailings.

Conclusions and next steps

Further analyses will determine background THg levels to calculate a pollution index. An age model and Hg isotope analysis are planned for the PNG samples to confirm whether contamination aligns with tailings disposal over the past 25 years, helping assess environmental risks in both regions.

Email contact

okeshk@geomar.de

Funding source

Base funding from EOMAR Helmholtz Centre for Ocean Research Kiel

AB Landrigan: Toxic Metals and Global Health - Lessons from the Lead Poisoning Pandemic

Philip J. Landrigan, MD, MSc - Boston College The United States of America (USA)

Dr. Landrigan is Director of the Global Observatory on Planetary Health at Boston College, where he and his team generate new scientific knowledge on the health impacts of climate change, pollution, and biodiversity loss – the three great interlinked planetary-scale challenges of our time – and translate this scientific knowledge into evidence-based and ethically grounded actions and policies that will slow climate change, prevent disease, advance social justice, reduce inequity, and save lives.

All authors and affiliations

Philip J. Landrigan, MD, MSc (1,2)

1. Global Observatory on Planetary Health, Boston College, Chestnut Hill, MA, USA
2. Center Scientifique de Monaco, Monaco, MC

Background or Purpose

Lead is an ancient metal that humans have mined, smelted and used for over 5000 years. Lead poisoning has been recognized for 2,000 years and was described in detail by Bernardino Ramazzini. Until the advent of mechanized mining, it was largely a disease of workers.

Content

Since the late 1800s, lead use has increased exponentially. Lead-based paint, tetraethyl lead for petrol, and lead-acid batteries have come to market. Global use continues to rise the world turns increasingly towards electricity. Widespread environmental contamination and extensive human exposure have resulted. Lead poisoning has moved beyond the workplace. Children are at particular risk.

A key development was the recognition that lead can cause poisoning in the absence of symptoms. In children, silent lead poisoning causes lifelong loss of cognitive function, shortened attention span and behavioural disruption. In adults, it increases risk for cardiovascular and renal disease. In 2019, lead was responsible for the loss of 765 million IQ points in children, 90% in low- and middle-income countries (LMICs), and for 5.5 million deaths among adults. The resulting annual economic losses are estimated to be $6.0 trillion (USD). It is now understood that no level of lead is safe.

Lead poisoning can be prevented by preventing lead exposure and is highly cost-effective. Removal of lead from petrol in the USA reduced children's mean blood lead level by 95%, increased the mean IQ of all children by 5 points, and created an annual economic benefit of $200 billion, an aggregate benefit since 1980 of over $8 trillion. Thanks to work by WHO and UNEP, lead has now been removed from automotive petrol in all countries worldwide.

Implications for addressing the issue

Despite much progress, the lead poisoning pandemic continues. One of 3 every children worldwide has an elevated blood level.

Potential follow-up/actions

We still have much work to do.

Email contact

phil.landrigan@bc.edu

AB Lu, JL.: ASGM (Artisanal Small Scale Gold Mining) and occupational health in the Philippines

Jinky Leilaie Lu - National Institutes of Health, University of the Philippines Manila, The Philippines

Jinky Leilanie Lu is a Research Professor at the National Institutes of Health, University of the Philippines Manila. She is also the Chair of the International Commission on Occupational Health-Mining Occupational safety and Health.

All authors and affiliations

Jinky Leilanie Lu (1); Sophia Francesca Chua (2)

1. National Institutes of Health, University of the Philippines Manila
2. Queensland University of Technology, Brisbane, Australia, PhD And.

Background

This comprehensive research report covers an overview of the land use in T'boli including mining areas which are the target sites of the study, and proceeds to present the results of the chemical contamination to the environment and community, particularly, mercury as a toxic chemical, using chemical analysis of four environmental media- soil, water, fish and plant/biota.

Methods/Approach

The mercury level in blood of miners and workers was also measured and presented in this report as evidence of possible human toxicity arising from mines exposures. The other hazards widely known and documented in literature including noise, vibration, extremes of temperature, and weather parameters were also measured using certain scientific equipment/ instruments.

Results

Based on German Human Biomonitoring (HBM) values: HBM-I = 0.005 µg/mL; HBM-II = 0.015 µg/mL; 8.13% of the209 blood samples of miners are in category HBM-II needing intervention. 43% are in between HBM -I and HBM-II which calls for confirmation for potential sources. PLANT QS based on Indonesian National Agency of Drug and Food Control Quality Standards (QS) = 0.5 mg/kg, 21.05% was used for plants, and of the 76 plant samples exceeded the standards. Based on the Philippine DENR (DAO-2016-08) General Effluent Standards 2016 Maximum Allowable Limit (MAL) = 0.004 mg/L for release to Class C water bodies, two percent of the 24 water samples exceeded the standards. Of the 30 samples of fish, only one exceeded the FISH MRL based on Philippine National Standards – Bureau of Agriculture and Fisheries Standards 2020 (Fish and fishery products – Tilapia) Maximum Residue Level (MRL) = 0.5 mg/kg. Above threshold limit values were also taken from air monitoring.

Conclusions and next steps

The study has shown results that are not within standard values for air, water, plant, fish, and human blood samples, evidencing the risks to health among small scale miners.

Email contact

jinky_lu@yahoo.com

AB Lu, Y.: Monitoring lead exposure in Indian children

Yi Lu – Vital Strategies – New York (USA)

Yi Lu is a senior program manager at vital strategies, New York. She is a collaborative, bilingual environmental epidemiologist with a strong medical background, passionate about global health issues and translating science into policy and practice.

All authors and affiliations

Yi Lu (1)

1. Vital Strategies, New York (USA)

Background

According to estimates from the GBD study, India accounted for more than half of the total global morbidity and mortality attributable to lead, despite having only 18% of the global population. Based on modelled estimates, over 25 Indian states had average BLLs above 5 ug/dL. However, statewide monitoring and data on lead exposure were not available to verify these estimates. We provide an overview of design considerations and differences in findings from our state-level blood lead surveillance among children in Bihar and Tamil Nadu states in India.

Methods/Approach

We initiated surveillance efforts in two Indian states in collaboration with Pure Earth, local governments, and research organizations. We summarized and compared key components of surveillance approaches, including design considerations and implementation challenges as well as findings from the two states including distribution of lead exposure and potential risk factors.

Results

Approaches to understanding lead exposure among Indian children need to be customized by states due to the large variation in geography, health service delivery systems, existing laboratory capacity, and human resources. We compared sources and risk factors for lead exposure observed across the two states and shared some unique challenges.

Email contact

ylu@vitalstrategies.org

Funding source:

This study was made possible by Pure Earth and GiveWell and was funded in part by the Effective Altruism Global Health and Development Fund, Open Philanthropy, and Affinity Impact.

AB Makris: The effect of an organic food intervention treatment on biomarkers of exposure to lead and cadmium in primary school children of Cyprus: A cluster-randomized crossover trial

Konstantinos C. Makris - Cyprus University of Technology Cyprus

Dr. Makris is a full professor of environmental health sciences applying the concept of the human exposome and its tools to better understand the development and testing of non-pharmacological health interventions towards reducing body burden of environmental chemicals, including metals.

All authors and affiliations

Shamah A Agboola (1); Corina Konstantinou (1); Pantelis Charisiadis (1); Thibaut Delplancke (1); Nikolaos Efthymiou (1); Konstantinos C Makris (1)

1. Cyprus International Institute for Environmental and Public Health, School of Health Sciences, Cyprus University of Technology, Limassol, Cyprus.

Background

Food contaminants, such as, pesticides and metals are ubiquitous in the food chain. Studies routinely report on the metals content of organic and conventional food crops. However, comparative human studies on the body burden of metals associated with organic food consumption are currently lacking.

Methods/Approach

The objectives were to i) determine the effectiveness of an organic food intervention in reducing the body burden of urinary concentration of lead (Pb) and cadmium (Cd) and ii) evaluate the association between metal exposures and biomarkers of oxidative damage in primary school children in Cyprus. This study was part of the ORGANIKO cluster-randomized crossover trial, a 40-day organic food treatment to 149 healthy children (10-12 years) in Cyprus. Urinary biomarkers of Pb and Cd were determined using inductively coupled plasma mass spectrometry. Linear mixed-effect regression models were used to account for the effect and duration of the organic food treatment. Multiple comparisons were handled using Benjamini-Hochberg correction.

Results

A time-dependent reduction for creatinine-adjusted Pb during the intervention period was observed ($\beta = -0.021$; 95% CI: -0.034, -0.008; p-adjusted = 0.01). A similar trend was observed for creatinine-adjusted Cd, but it was not significant ($\beta = -0.013$; 95% CI: -0.026, 0.000; p-adjusted = 0.15). The creatinine-adjusted oxidative damage biomarkers were significantly associated with both metal biomarkers; Pb (8-OHdG: $\beta = 0.388$, 95% CI: 0.303, 0.472; p-adjusted<0.001).

Conclusions and next steps

A 40-day organic food treatment reduced primary school children's exposures to Pb over time. More human studies in settings with high food contaminant profiles across common crops are warranted.

Email contact

konstantinos.makris@cut.ac.cy

Funding source

ORGANIKO LIFE

AB Manhart: Used Lead-acid Battery Recycling in Africa and India

Andreas Manhart - Oeko-Insitut, Germany

Andreas Manhart is Senior Researcher at Oeko-Institut on raw materials, circular economy and recycling. His work focuses on socioeconomic, technical and regulatory framework conditions and drivers enabling or hindering sustainable raw material cycles. He is engaged in various research and implementation projects in the EU and African contexts with key foci on complex and hazardous waste types such as batteries.

All authors and affiliations

Andreas Manhart (1); Anuradha Varanasi (2)
1. Oeko-Institut
2. Visiting Researcher Oeko-Institut

Background or Purpose

Note: we designed an abstract and split this in 3 sections (the 3 below). We think that this should be one consolidated text-block:
The use of lead-acid batteries is growing in almost all low- and middle-income countries. Due to the high material value of lead and their average lead content of around 60-65%, the batteries are typically collected for recycling. The lead-acid battery recycling landscapes in low- and middle- income countries have developed significantly over the last decade and are now characterized by large, registered recycling facilities equipped with metallurgical furnaces and lead refinery lines. Nevertheless, many of these plants operate at sub-standard in terms of health and safety and pollution control. They are therefore a significant source of lead emissions to the workplace and the environment. This presentation will give insights into lead-acid battery recycling landscapes in selected African countries as well as India.

Content

The use of lead-acid batteries is growing in almost all low- and middle-income countries. Due to the high material value of lead and their average lead content of around 60-65%, the batteries are typically collected for recycling. The lead-acid battery recycling landscapes in low- and middle- income countries have developed significantly over the last decade and are now characterized by large, registered recycling facilities equipped with metallurgical furnaces and lead refinery lines.

Implications for addressing the issue

Nevertheless, many of these plants operate at sub-standard in terms of health and safety and pollution control. They are therefore a significant source of lead emissions to the workplace and the environment.

Potential follow-up/actions

This presentation will give insights into lead-acid battery recycling landscapes in selected African countries as well as India with a view to generate entry points for improvement strategies.

Email contact

a.manhart@oeko.de

AB Mufune: Childhood Anaemia and Malnutrition Correlated to Lead Exposure in Zambia

Tiza Mufune – Ludwig Maximilian University of Munich Germany

Dr. Tiza Mufune is a District Medical Officer at the Ministry of Health in Kabwe, where he has worked with the co-authors to understand the sources, distribution and effects of lead exposure among

affected populations in Kabwe Town, Zambia. He is also a PhD candidate at the Ludwig Maximilian University of Munich – Centre for International Health.

All authors and affiliations

Tiza Mufune (1,2); John Yabe (3,4); Seter Siziya (5); Ursula Berger (6); Dennis Nowak (7); Given Moonga (1,9); David Mulemena (8); Stephan Bose-O'Reilly (7)

1. Center for International Health, Ludwig-Maximilians University, Munich, Germany
2. Ministry of Health, Kabwe District Health Management Team, Kabwe, Zambia
3. School of Veterinary Medicine, The University of Zambia, Lusaka, Zambia
4. School of Veterinary Medicine, University of Namibia, Windhoek, Namibia
5. School of Medicine, Copperbelt University, Kitwe, Zambia
6. Ludwig -Maximilians University Hospital, Institute of Biometrics and Epidemiology, Munich, Germany
7. Institute and Clinic for Occupational, Social and Environmental Medicine, University Hospital, Ludwig-Maximilians University, Munich, Germany
8. USAID Zambia, Accessible Markets for Health, John Snow Inc, Research and Training Institute, Lusaka, Zambia
9. School of Public Health, University of Zambia, Lusaka, Zambia

Background

Environmental lead contamination in Kabwe Zambia, is a long standing legacy resulting from mining activities. This presents a serious public health issue, especially among young children. In this study, we sought to establish the relationship between lead exposure and anaemia as well as nutritional status in children living in areas with elevated lead levels.

Methods/Approach

A cross sectional study was conducted among children aged zero to 15 years (n=10,718) residing in four townships. Data on demographic characteristics, anthropometric measurements, blood lead levels (BLLs) and blood haemoglobin (Hb) were collected. Associations were determined using chi square test, and binary logistic regression was conducted. The results are reported as odds ratios (ORs) and their 95% confidence intervals (95% CIs).

Results

The median age was 7.3 years (Interquartile range [IQR] 3.9, 10.7), the median BLL was 22.4µg/dL (IQR 13.9, 34.1) the mean Hb was 12.3g/dL (95% CI: 12.13, 12.36) and the prevalence of anaemia was 23.4%. The findings indicated a statistically significant association between younger age and elevated BLL. Makululu Health Center exhibited a substantially higher likelihood of children having a BLL exceeding 45µg/dL compared to Chowa Health Center (OR: 1.76; 95% CI: 1.44, 2.16). Conversely, Katondo Health Center showed a lower likelihood for children to have BLLs above 45µg/dL, compared to Chowa Health Center (OR 0.10; 95% CI: 0.07, 0.16). Furthermore, children with low height for age had a higher likelihood of having a BLL above 45µg/dL, compared to their normal counterparts (OR 1.42; 95% CI: 1.17, 1.71). Similarly, low Hb in children increased their likelihood of having a BLL above 45µg/dL (moderately low Hb: OR 1.52; 95% CI: 1.25, 1.83; low Hb OR 1.96; 95% CI: 1.39, 2.75).

Conclusions and next steps

Children residing near the old lead mine in Kabwe, particularly those with anemia and stunting, should be routinely screened for lead exposure.

Email contact

mufunetiza@yahoo.com

Funding source

Funding source was from the World Bank Group, through the Zambia Mining and Environmental Remediation and Improvement Project

AB Musa Obadia: Occupational metal exposure and health outcomes in the Congolese copper belt region

Musa Obadia Paul - University of Lubumbashi, The Democratic Republic of Congo

Dr. Musa Obadia Paul is the Chairperson of the Poison Control Centre at the University of Lubumbashi in the Democratic Republic of Congo. A physician and public health researcher, he leads efforts in the study of health effects related to exposure to toxic substances, with a particular focus on heavy metals and environmental pollutants.

All authors and affiliations

Musa Obadia Paul (1); Pyana Kitenge Joseph (1); Mundali Shimbi Desiré (1); Banza Lubaba Célestin (1); Benoit Nemery (2)

1. University of Lubumbashi, Lubumbashi, DR Congo
2. KU Leuven, Leuven, Belgium

Background

Workplace metal exposure poses significant health risks. We conducted four studies examining its effects: exposure levels in smelter workers, erectile dysfunction in artisanal miners, respiratory health in refinery workers, and behavioural outcomes in child miners, offering a broad perspective on metal-related health impacts.

Methods/Approach

We conducted four cross-sectional studies to assess health risks related to trace metal exposure. We included smelters, samplers, and controls to assess trace metals in blood and urine (ICP-MS). Erectile function, hormone levels, and marital quality were compared between miners and bakers. Respiratory symptoms and spirometry were assessed in refinery workers. In child miners, behavioural screening and urinary metals were compared between miners and farming peers.

Results

Smelter workers showed higher blood cobalt (0.82 µg/L) and lead (190 µg/L) than samplers and controls, and elevated urinary cobalt (10.7 µg/g creatinine), germanium (0.15), and lead (9.51).

Artisanal miners had lower IIEF scores (66 vs. 73), lower free testosterone (8.11 vs. 10.52 ng/dL), and greater odds of erectile dysfunction (aOR, 2.6; 95% CI, 1.3-5.3). Refinery workers had high levels of cobalt, arsenic, and lead; dyspnoea was more frequent in lixiviation. Child miners had 4.7 times higher urinary cobalt and 2.6 times more manganese than controls, with more hyperactivity and peer problems.

Conclusions and next steps

Trace metal exposure is high in several occupational groups in the Copperbelt region, with documented effects on sexual, respiratory, and behavioural health. These findings call for urgent occupational and public health interventions to reduce exposure and protect vulnerable groups.

Email contact

musa.p.obadia@gmail.com

Funding source

Ares-CUD, Vlir UOS, Belgian organisations

AB Rakete: Microsampling as potential tool for toxic metal assessment

Stefan Rakete - LMU University Hospital, Germany

Dr. Stefan Rakete is analytical chemist with special interest in the development of innovative methods for toxic metal analysis in human and environmental samples.

All authors and affiliations

Stefan Rakete (1)

1. Institute and Clinic for Occupational, Social and Environmental Medicine, LMU University Hospital, LMU Munich, Munich, Germany

Background

The exposure to toxic metals is associated with many adverse health effects including cancer and neurological diseases. Monitoring of toxic metal levels in biological matrices such as blood is one of the key elements for exposure assessment. However, commonly applied venous blood sampling comes with many disadvantages, e.g., relatively high costs due medical personnel as well as cooling and shipment restrictions. Microsampling-assisted monitoring of toxic metals in blood comes with the promise of simplification and wider availability of exposure assessment. The content of presentation is a brief overview about current microsampling techniques and their applications for toxic metal analysis in blood.

Methods/Approach

The existing literature was reviewed for studies applying microsampling for toxic metal biomonitoring using scientific databases.

Results

Sampling devices such Dried Blood Spots (DBS) or Volumetric Absorptive Microsampling (VAMS) have

been used for toxic metal analysis in blood. Major challenges including sensitivity, reproducibility and background contamination of the sampling material were identified. Only a few studies have applied microsampling for biomonitoring of toxic metals to assess occupational or environmental exposure to toxic metals. Furthermore, microsampling methods have yet not been included in regular biomonitoring programs.

Conclusions and next steps

Although the research and use of microsampling-assisted toxic metal biomonitoring is intensifying, standardization and field applicability of these methods still need to be demonstrated. Nevertheless, these tools have the potential support and improve exposure assessment, especially in low- and middle-income countries.

Email contact

stefan.rakete@med.uni-muenchen.de

AB Rösch: Genotoxicity testing with heavy metals – rolling the dice?

Christine Rösch - CFCS-Consult GmbH Germany

Dr. Christine Rösch is Senior Toxicologist with long-time experience in experimental toxicology. For REACH purposes, she is supporting companies in registering their compounds containing metals or heavy metals, respectively.

All authors and affiliations

Christine Rösch (1); Ricarda Rohrig (1); Jutta Fuhlrott (1)

1. CFCS-Consult GmbH

Background

REACH regulation (1907/2006 (EU)) demands genotoxicity testing for substances depending on the yearly tonnage band manufactured in the EU. Unfortunately, in vitro genotoxicity testing of metals turns out to be challenging due to methodological limitations and inconsistent predictive outcomes. While standard in vitro assays are designed rather for organic compounds, testing (heavy) metals or metal compounds presents unique challenges due to their chemical properties and interactions with biological systems.

Methods/Approach

Standard bacterial assays like the Ames test often yield false negatives for heavy metals due to the inability of metal cations to permeate bacterial cell walls effectively. The standard in vitro mammalian cell tests demanded by REACH, like the micronucleus test or the chromosome aberration assay, exhibit rather poor specificity and are prone to artifacts. For example, some cell lines are deficient in DNA repair. Also, cell lines do not consider ADME factors: uptake, distribution and metabolism of metals differ fundamentally from organic molecules. Some metals require special transport mechanisms or have an indirect genotoxic effect, which is not well reflected in standard tests.

Genotoxicity testing in vivo does not guarantee unequivocal results, either. Testing at excessively high concentrations, which are unrealistic to achieve in animals and humans under normal physiological conditions, may trigger nonspecific cytotoxicity or stress response. Thus, their outcome may be of doubtful relevance for humans.

Results

Here, as a practical example, the complexity of genotoxicity testing shall be demonstrated based on results obtained with an organometallic compound of copper.

Conclusions and next steps

In conclusion: when genotoxicity testing becomes necessary during REACH registration of your compound containing metals, it is crucial to evaluate any potential pitfalls beforehand and design your testing strategy as specific as possible to avoid producing results that may not accurately reflect the true genotoxic or non-genotoxic properties of your substance.

Email contact

roesch@cfcs-consult.de

AB Sargsyan: Rapid market screening - lead in consumer products

Aelita Sargsyan - Pure Earth, The United States of America (USA)

Dr. Aelita Sargsyan is a Technical Advisory Board member with Pure Earth (formerly Blacksmith Institute) and a Doctoral student in the Programme in Toxicology, Pollution and Environmental Health at the University of Valencia.

All authors and affiliations

Aelita Sargsyan (1,2); Emily Nash (1); Gordon Binkhorst (1); Jenna E. Forsyth (3); Barbara Jones (4); Gabriel Sanchez Ibarra (1); Sarah Berg (1); Andrew McCartor (1); Richard Fuller (1); Stephan Bose-O'Reilly (1,5)

1. Pure Earth, 475 Riverside Drive, New York, NY 10115, USA
2. Doctoral Program in Pollution, Toxicology and Environmental Health, Faculty of Biological Sciences, University of Valencia, c/Dr. Moliner, 50, Burjassot, 46100 Valencia, Spain
3. Division of Infectious Diseases and Geographic Medicine, Stanford University, Stanford, CA, USA
4. Cardinal Resources, Inc., 4410 Broadway Blvd., Monroeville, PA 15146, USA
5. Institute and Clinic for Occupational, Social and Environmental Medicine, University Hospital, LMU Munich, Ziemssenstr. 5, 80336 Munich, Germany

Background

Lead poisoning remains a major but underrecognized public health threat, particularly in low- and middle-income countries (LMICs). Around the world, lead exposure originates from diverse sources, including everyday household items such as foodware and spices, as well as industrial pollution. This

study aimed to investigate whether common consumer goods in LMIC markets contain lead at levels that could plausibly contribute to exposure.

Methods/Approach

In 25 countries, research teams systematically collected and tested 5,007 products—including metal and ceramic foodware, cosmetics, toys, paints, spices, and staple foods—using portable X-ray fluorescence (XRF) analyse rs. A subset of samples was tested via ICP-MS laboratory analysis in the USA.

Results

Results revealed that 51% of metal foodware, 45% of ceramic foodware, and 41% of paints exceeded international reference thresholds of lead concentrations. Some traditional cosmetics, such as kohl/kajal, showed extreme concentrations, with lead levels exceeding 600,000 ppm.

Conclusions and next steps

The findings highlight the widespread presence of hazardous products and point to urgent needs for enforcement, consumer protections, and scalable field-testing tools. Rapid Market Screening (RMS) offers a practical, low-cost method to quickly identify high-risk items and inform targeted interventions to reduce lead exposure globally.

Email contact

aelita@pureearth.org

Funding source

RMS was made possible by GiveWell, funded in part by Effective Altruism Global Health and Development Fund, Open Philanthropy, and Affinity Impact

AB Sen: Strategies for safeguarding workers from toxic metals exposure at brown-field mineral & metals projects

Dr. Krishna Nirmalya Sen - Larsen & Toubro Limited, Minerals & Metals, India

Dr Krishna Nirmalya Sen is the Head of Environment, Health and Safety Dept of Minerals & Metals IC of Larsen & Toubro Limited. He also volunteers as the Chair of OHS Expert Committee of Indian Chamber of Commerce and President of ASSP India Chapter. He is a Fellow of Collegium Ramazzini and Institution of Engineers India.

All authors and affiliations

Krishna Nirmalya Sen (1); Gollapalli Muralidhar (2)

1., 2. Larsen & Toubro Limited, Minerals & Metals IC, Kolkata, India

Background

Projects related to modernization, expansion and repair in the mineral and metals industry poses significant occupational health and safety (OHS) risks to the workers due to intrinsic nature of the activities and work environments.

Methods/Approach

Challenges posed by brownfield expansion projects require comprehensive, proactive safety frameworks beyond traditional compliance-based approaches. In this paper, efforts made to highlight structured safety framework adopted to ensure safe and healthy workplaces in line with Vision Zero philosophy. The approach encompasses majorly on three main areas, namely, Safe Processes, Competent Workmen and Application of Digital Technologies.

Results

Implementation of integrated strategy for brownfield projects leads to measurable reductions in safety and health incidents, improved compliance, and greater worker wellness through participation and engagement. Adoption of vision Zero principles fostered a culture of accountability and continuous improvement. Collectively, this integrated approach resulted in smooth handling of complex expansion jobs without compromising safety and health of employees.

Conclusions and next steps

OHS risk management and control associated with construction work of brownfield expansion and modernization of minerals and metals plants, requires multipronged approach. Leadership commitment integrated with establishment of safe processes, workmen competency and technological solution is the way for future.

Email contact

knsen@lntecc.com

AB Sibanda: An investigation on the adverse impacts of mineral mining on the environment. (A case study of Zimbabwe)

Brandon Phathisani Sibanda - Enviro recsus, Zimbabwe

Mr Brandon Phathisani Sibanda is the environmental scientist at Rusununguko Nkululenko holdings and has assisted the Zimbabwe National Army in its mining projects across Zimbabwe, in an effort to promote environmental rehabilitation and reduce environmental degradation as a result of mineral mining.

All authors and affiliations

Brandon Phathisani Sibanda (1); Tawanda Chaukura (2)

1. Midlands state university, Gweru, Zimbabwe and environmental scientist at environmental rescus Harare, Zimbabwe
2. Tawanda Chaukura University of Zimbabwe, Mining engineering department, Harare, Zimbabwe

Background or Purpose

Investigating on the polices that are contributing to environmental degradation in Zimbabwe. These policies will in turn promote activities that aren't environmentally sustainable and degrade the environment. The role of investors is also in the spot light as they also are part and parcel of the network as they finance the activities, a closer look at the investors it also has to be proven if they are environmentally conscious in nature. The countries legislation on activities that impact the environment is also examined to figure out if they are in line with sustainable environment development. The countries communities is also questionable on the level of environmental awareness as there is also small scale activities in communities that degrade the environment and exposure to toxins.

Content

Zimbabwean environmental legislation.
Investors activities in the country.
Mineral composition of the country.
Small scale mining activities.
Investigation of toxic metals exposure.

Implications for addressing the issue

No direct implications however most of the mining activities are being done by Chinese nationals hence limited implications on the country’s government.

Potential follow-up/actions

Education at community level on the impacts of toxic metals exposure.

Email contact

brandonpsibbz@gmail.com

Funding source

Germany research foundation

AB Smith: New analytical approaches to inform exposure assessment and treatment modalities

Don Smith - University of California, Santa Cruz The United States of America (USA)

Dr. Smith is a Distinguished Professor of Microbiology and Environmental Toxicology at the University of California, Santa Cruz, where he conducts research in environmental health, including exposures and neurotoxicology of environmental agents, exposure pathways to susceptible human populations such as children, the neuromolecular mechanisms underlying neurotoxicity, and the efficacy of therapeutics for the treatment of metal toxicity.

All authors and affiliations

Don Smith (1)

1. University of California, Santa Cruz, California, USA

Background

Accurate assessment of environmental exposures to toxic metals remains the Achille's heel of environmental health studies seeking to determine the adverse health effects those exposures. Further, the identification and validation of exposure biomarkers is fundamental to human toxicology and risk assessment.

Methods/Approach

Over the past decade, we have developed and/or advanced analytical approaches to improve exposure assessment and risk for adverse health effects from a variety of metals, including lead, manganese, and arsenic. For lead, we have advanced stable lead isotope methods to identify exogenous and endogenous sources of exposure to children, adults, and critically endangered wildlife. For manganese, we have developed laser-ablation-inductively coupled mass spectrometry (LA-ICP-MS) methods to determine foetal and postnatal childhood manganese exposure using shed deciduous teeth, and to develop the use of human hair as an integrative biomarker of environmental manganese exposure.

Results

Our studies have shown that 1) lead accumulated in the skeleton poses a significant endogenous source of exposure, potentially stymieing the efficacy of environmental remediation efforts, and 2) retained/embedded lead fragments, such as often arises as a consequence of non-fatal firearm injuries, produces an endogenous source of prolonged elevated lead exposure that stymies the efficacy of therapeutic chelation treatment to reduce body lead burdens and toxicity. Similarly, our studies with manganese have shown that the adverse health effects associated with elevated manganese exposure in children and adolescents differ depending on pre- vs postnatal exposure windows of susceptibility.

Conclusions and next steps

Altogether, these studies underscore the continued need for the development and advancement of exposure and health effect assessment methods.

Email contact

drsmith@ucsc.edu

Funding source

US National Institute of Environmental Health Sciences, UC Santa Cruz, US Veterans Administration

AB Straif: The Carcinogenicity of Metals

Kurt Straif - Boston College & ISGlobal, Spain

Kurt Straif is trained in oncology, occupational, environmental and social medicine, public health and epidemiology. He served as the senior epidemiologist, Head of the IARC Monographs and Head of the IARC Section of Evidence Synthesis and Classification (overseeing the WHO Classification of Tumours, and IARC Handbooks of Cancer Prevention). Since retirement from IARC he continues his work on global cancer prevention at Boston College and ISGlobal, plus a focus on climate change and health.

All authors and affiliations

Kurt Straif (1)

1. Boston College, MA, USA

ISGlobal, Barcelona, Spain

Background

The mining of minerals has played a significant role in human history, for human development, but unfortunately also in terms of massive occupational and environmental disasters, from serious injuries and frequent premature deaths among miners to increased cancer risks, long-term environmental pollution in the vicinity of large and small-scale mining activities, and finally in the recycling of precious metals.

Methods/Approach

Over the centuries, and particularly since the second half of the last century, various occupational exposures in mining have been identified as human carcinogens.

Results

This overview will focus on the human carcinogenicity of various metals, building on the evaluations by the IARC Monographs programme, and with an emphasis on the human evidence by exposure to selected metals, specifying the evidence by cancer site.

Conclusions and next steps

Still, new research gaps emerge from the latest consensus evaluations, and new evidence supported the recommendation of some metals for future priority evaluations by the IARC Monographs programme.

Email contact

AB Tetsopgang: Estimation of mercury released into the environment from the uncontrolled dumping of broken medical thermometers in hospitals in Cameroon, Africa

Samuel Tetsopgang - Association Institute of Total Environment (INTEV), Cameroon

Dr. Samuel Tetsopgang is the President and Co-Founder of Association Institute of Total Environment (INTEV), created and based in Yaoundé, Cameroon. INTEV is accredited to UNEP since 2022. INTEV carried out research and advocate before decision makers on toxic chemicals (and precisely heavy metals) on products.

All authors and affiliations

Samuel Tetsopgang (1)

1. Association Institute of Total Environment (INTEV), P.O. Box 31314 Yaoundé, Cameroon

Background

Mercury is a neurotoxic element. Owning to its unique physical and chemical properties, mercury is present in several daily use devices and will be released into the land and waters after the end of life or through mishandling. There is a permanent usage of mercury thermometers in hospitals and homes because of malaria prevalence in some countries. Then, the accidental breakage of these thermometers will increase the budget of mercury emitted to land and waters. The main objective of this survey was to estimate the quantity of mercury released into the environment through the uncontrolled dumping of broken thermometers in hospitals in Cameroon.

Methods/Approach

In fact, each patient is required to hold a thermometer for any health consultation in Cameroon. Then a questionnaire was sent out to assess thermometers handled by each medical personnel in respect to categories, total units and broken units during medical consultations. Medical personnel responding to questionnaires comprised assistant nurses (AN), nurses, midwives and physicians. The total of broken mercury thermometers was used to quantity mercury yearly, accidently released into the environment.

Results

This survey indicates that mercury added thermometers are the most common with 2736 units used for a period of a year. Then, there is a yearly disposal of 0.168 to 0.504 Kg of mercury by AN in

hospitals in Yaoundé. Then, the total national Hg disposed of from the uncontrolled dumping of broken mercury-added thermometers is estimated to 222.52 Kg in Cameroon.

Conclusions and next steps

The total estimation of 0.223 Kg of mercury disposed of in this study is a very small portion of the 2220 tonnes of annual global anthropogenic mercury emissions. Then, this estimation turns out to be significant in the local level since it makes about 8.58 % of mercury use in ASGM in Cameroon.

Email contact

tetsopgang@yahoo.com

Funding source

Funding was received from the Association Institute of Total Environment (INTEV) for field works to carry out this study

AB Varanasi: Working Towards Zero Lead Emissions From the Battery Recycling Industry For A Just Energy Transition in India

Anuradha Varanasi – Oeko-Institut Germany

Anuradha Varanasi is a visiting researcher at Oeko-Institut and a freelance science journalist based in Freiburg Germany. She covers climate change and renewable energy and her stories have been published in Atlas Obscura, Popular Science, Inverse, Wired, Cipher News, and other news publications.

Background or Purpose

While India has set an ambitious target of installing 500 gigawatts of renewable energy by 2030, it will need more battery storage than any other country. Lead-acid batteries reign supreme in India for storing electricity because they are 40% to 50% cheaper than lithium-ion batteries. Additionally, the country's thriving three-wheeler electric vehicles industry also predominantly relies on this low-cost technology.

As India's ambitious green energy transition is rapidly underway, the use of lead-acid batteries is growing. However, this rising demand threatens to overwhelm India's unregulated battery recycling sector.

Content

Environmentally unsound lead smelters and battery recyclers — which are the most polluting industry worldwide — are a major source of lead pollution in 23 states across India. Despite that, the Central and State Pollution Control Boards have repeatedly failed to enforce regulations. Children are

particularly vulnerable to acute lead poisoning that is proven to cause behavioural and intellectual disabilities. Of the 800 million children with elevated blood lead levels globally, 275 million live in India. Without systemic change, extremely high levels of lead emissions from unsound battery recycling plants will wreak havoc on public health. Intergenerational lead exposures have kept marginalised communities trapped in a cycle of poverty.

Implications for addressing the issue

The Indian lead industry has become a global hub that supplies raw materials to European markets involved in the green energy transition. My research focuses on solutions from successful sector transitions towards well-controlled lead battery recycling (as experienced in Germany) and evaluating how those solutions could be transferred to developing countries like India.

Potential follow-up/actions

My research project aims to document this environmental justice issue through solutions-oriented news articles. My fieldwork in two Indian states (Bihar and Maharashtra) has enabled me to gather exclusive information on battery waste management systems in India.

Email contact

asv2137@columbia.edu

Funding source

Alexander von Humboldt Foundation

AB Velasco-Garrido: High lead exposure related to intake of Ayurvedic pharmaceuticals

Marcial Velasco Garrido, Universitätsklinikum Hamburg-Eppendorf

All authors and affiliations Marcial Velasco Garrido, Stefanie Selke, Volker Harth, Alexandra M. Preisser

Universitätsklinikum Hamburg-Eppendorf

Background or Purpose

A 47-year-old woman presented to the environmental medicine outpatient clinic of the Institute for Occupational and Maritime Medicine in 02/2023 after a two-month stay in an Ayurvedic clinic in India. After her stay at the clinic, during which she had undergone Ayurvedic therapy, she imported various products to Germany and continued to take them. In the meantime, she was concerned about possible exposure to heavy metals. The medical history revealed no evidence of manifest heavy metal intoxication.

Results

As a precautionary measure, we carried out human biomonitoring (HBM) for arsenic (As), lead (Pb) and mercury (Hg). In addition, we analysed the pharmaceutical products brought by the patient. The heavy metal content of the products was determined by Inductively Coupled Plasma Mass Spectrometry after aqua regia digestion and incubation in dilute nitric acid, dilute hydrochloric acid and thiourea.

Lead levels were 713 µg/L (Ref. = 30 µg/l) in blood and 88.8 µg/L (Ref. = 30 µg/l) in urine. Mercury was slightly elevated in urine 1.49 µg/l (Ref. = 1.0 µg/l). Mercury blood levels and arsenic urine levels were within their respective reference values. The blood count showed mild anaemia (Hb 11.9 g/dl). Serum creatinine, glomerular filtration rate and liver enzymes were within normal range. We recommended

immediate discontinuation of the ayurvedic medication and further regular control of lead blood levels. The analysis of the ayurvedic products brought by the patient (n= 6) revealed that the maximum level according to EU 2023/915 (‘Contaminants Regulation’) for lead (3.0 µg/g) was exceeded in three of the products: ‘Stresan’ (pill) = 3.04 µg/g, ‘Ashwaganda Pak’ (granulate) = 2703.9 µg/g and ‘Maanasa Mithra-Vatakam’ (pill) = 78.47 µg/g. The maximum level for mercury (0.1 µg/g) was exceeded in 5 preparations (Hg content between 0.14 µg/g and 1096 µg/g).

Conclusions and next steps

Despite public warnings about the hazards associated with Ayurvedic pharmaceuticals, these products continue to be consumed. In this case, the patient’s initiative to seek advice and evidence-based counselling prevented the development of a symptomatic intoxication with lead.

Email contact

m.velasco-garrido@uke.de

AB Vukelic: Benchmark Dose Analysis of Lead Toxicity in Wistar Rats: Identifying Critical Toxic Effects

Dragana Vukelic - Department of Toxicology "Akademik Danilo Soldatović", University of Belgrade – Faculty of Pharmacy Serbia

Dr. Vukelic is a Regulatory Toxicologist at BASF-SE. She completed her doctoral research at the Department of Toxicology "Akademik Danilo Soldatović", University of Belgrade – Faculty of Pharmacy where she worked on several scientific projects focusing on the toxic metals. Her work included in vivo and in silico studies, with emphasis on Benchmark dose modelling to identify dose-response and critical effects of metal exposure.

All authors and affiliations

Dragana Javorac (1); Milena Anđelković (2); Aleksandra Buha Djordjevic (1); Evica Antonijević Miljaković (1); Katarina Baralić (1); Djurdjica Marić (1); Danijela Đukić-Ćosić (1); Marijana Ćurčić (1); Biljana Antonijević (1); Zorica Bulat (1)

1. Department of Toxicology “Akademik Danilo Soldatović”, University of Belgrade – Faculty of Pharmacy, 11221 Belgrade, Serbia
2. Health center Kosovska Mitrovica, 38220 Kosovska Mitrovica, Serbia

Background

Although lead (Pb) toxicity has been extensively studied over decades, recent concerns have emerged regarding the effects of chronic low-dose exposure, which may induce subtle and previously underrecognized biological alterations. This study investigates the critical toxic effects of lead (Pb) exposure on various organs and organ systems using a Wistar rat model.

Methods/Approach

The rats were divided into 7 groups of 6 animals, including one control group and 6 experimental groups treated with increasing doses of lead (0.1, 0.5, 1, 3, 7, 15 mg Pb/kg body weight/day) for 28

days. Twenty-four hours after the last dose, the rats were humanely sacrificed, and blood and organs were collected for further analysis. Blood samples were analysed for haematological parameters, biochemical markers, hormones, oxidative stress parameters, bioelements, and Pb content. Organs underwent histopathological examination, and oxidative stress markers, bioelement levels, Pb content, and brain acetylcholinesterase activity were measured.

Results

The most sensitive toxic effect of lead was a reduction in testosterone levels, with a benchmark dose lower confidence limit (BMDL) of 1.07e-06 mg Pb/kg body weight/day. Other critical effects had BMDLs up to approximately 0.1 mg Pb/kg body weight/day and included a decrease in copper (Cu) levels in blood and liver, inhibition of superoxide dismutase (SOD) in kidneys, and an increase in malondialdehyde (MDA) in the heart, reflecting lipid peroxidation. Elevated total oxidant status (TOS) in the brain and increased zinc (Zn) levels in the testis and pancreas were also identified as critical toxic effects.

Conclusions and next steps

The study highlights the importance of identifying critical toxic effects of Pb and provides insights into its toxicity mechanisms. Our results offer valuable data for human health risk assessment and evaluation of reference points for Pb. Further research will help health professionals make evidence-based decisions and improve environmental safety standards to protect human health and wellbeing.

Email contact

dragana.vukelic92@gmail.com

Funding source

This research was funded by the Ministry of Science, Technological Development and Innovation, Republic of Serbia through two Grant Agreements with Un

AB Yabe: Southern Africa - One health and toxic metals

John Yabe - University of Namibia, Namibia

Dr. John Yabe is a Senior Lecturer in Veterinary Pathology at the University of Namibia. He graduated with a PhD in Veterinary Pathology (Lead Poisoning) at Hokkaido University, Japan. He has been researching childhood lead poisoning for over ten years and is ranked among the Top 0.05% World Experts on Lead (https://expertscape.com/ex/lead). He is the Regional Coordinator for the JICA-Japan Project on heavy metal contamination in Zambia, Zimbabwe, Namibia, and Botswana (ZA.ZINAMBO Project).

All authors and affiliations

1. University of Namibia, Windhoek, Namibia
2. Hokkaido University, Sapporo, Japan

3. University of Zambia, Lusaka, Zambia
4. Ministry of Health, Kabwe, Zambia
5. Ministry of Fisheries and Livestock, Kabwe, Zambia

Background

Southern Africa is endowed with rich mineral deposits, where widespread environmental pollution with toxic metals has been reported. Kabwe town, in Zambia, has a long history of lead and zinc mining and serves as an example of the environmental impact of mining activities. Dense fumes rich in lead, cadmium, etc., polluted the environment in the town, which is ranked among “the ten worst polluted places on earth”.

Methods/Approach

A wide survey was conducted in Kabwe for laboratory screening of blood, milk, fecal, and urine samples in various townships.

Results

Findings from the University of Zambia and Hokkaido University collaborative study (KAMPAI Project; 2016-2022) revealed that animals and humans in communities in the vicinity of the mine were exposed to alarming levels of lead in Kabwe. The study analyse d the extent of lead poisoning and exposure variations among family members in Kabwe. The study also assessed Pb exposure and maternal characteristics on neurodevelopmental (ND) outcomes in children, as well as the clinical biochemical parameters associated with exposure to multiple environmental metals.

Conclusions and next steps

Following on from studies in Zambia, the researchers are launching a new project in Zambia, Zimbabwe, Namibia, and Botswana (ZAZINAMBO Project), from June 2025 to 2030, to extend the studies on the impact of toxic metals in the Southern African region. This will focus on One Health and Capacity Building.

Email contact

mjyabe@yahoo.co.uk
